# Rapid and minimally invasive preimplantation genetic testing for aneuploidies (PGT-A) based on polar body and nanopore sequencing: a viable alternative to conventional trophectoderm-based PGT-A?

**DOI:** 10.1093/hropen/hoaf069

**Published:** 2025-10-30

**Authors:** Di Song, Taoli Ding, Tuan Li, Peng Zhang, Yangyun Zou, Yuanbo Hu, Hong Ye, Yajun Xu, Shengnan Wang, Tuanping Zhou, Sijia Lu, Hongli Yan

**Affiliations:** Reproductive Medicine Center, The First Affiliated Hospital of Naval Medical University, Shanghai, China; Co-Innovation Center, Yikon Genomics Co., Ltd., Suzhou, China; Co-Innovation Center, Yikon Genomics Co., Ltd., Suzhou, China; Co-Innovation Center, Yikon Genomics Co., Ltd., Suzhou, China; Co-Innovation Center, Yikon Genomics Co., Ltd., Suzhou, China; Reproductive Medicine Center, The First Affiliated Hospital of Naval Medical University, Shanghai, China; Reproductive Medicine Center, The First Affiliated Hospital of Naval Medical University, Shanghai, China; Reproductive Medicine Center, The First Affiliated Hospital of Naval Medical University, Shanghai, China; Reproductive Medicine Center, The First Affiliated Hospital of Naval Medical University, Shanghai, China; Reproductive Medicine Center, The First Affiliated Hospital of Naval Medical University, Shanghai, China; Co-Innovation Center, Yikon Genomics Co., Ltd., Suzhou, China; Reproductive Medicine Center, The First Affiliated Hospital of Naval Medical University, Shanghai, China

**Keywords:** preimplantation genetic testing for aneuploidies (PGT-A), polar body (PB), nanopore sequencing, meiotic abnormalities, fresh embryo transfer

## Abstract

**STUDY QUESTION:**

Can third-generation sequencing (TGS)-based polar body (PB) analysis serve as a viable alternative to conventional trophectoderm (TE) biopsy for preimplantation genetic testing for aneuploidy (PGT-A)?

**SUMMARY ANSWER:**

This study demonstrates the feasibility of using TGS and PB biopsy for clinical PGT-A, particularly in advanced maternal age (AMA) patients.

**WHAT IS KNOWN ALREADY:**

TE biopsy, the current standard approach for PGT-A, is limited by embryonic mosaicism. Mosaicism potentially leads to false-positive aneuploidy diagnoses, resulting in the discard of genetically normal embryos and compromising the cumulative live birth rates (CLBRs).

**STUDY DESIGN, SIZE, DURATION:**

A total of 125 oocytes were collected from 30 couples. First (PB1) and second (PB2) polar bodies from 89 oocytes were individually amplified and sequenced, while those from the remaining 36 oocytes were processed jointly (PB1 + PB2). Then, 74 oocytes were successfully fertilized and developed into blastocysts (59.2% blastulation rate), and from these, corresponding TE biopsies were obtained.

**PARTICIPANTS/MATERIALS, SETTING, METHODS:**

Both PB and TE samples underwent whole-genome amplification (WGA) using multiple annealing and looping-based amplification cycles (MALBAC), followed by next-generation sequencing (NGS; all samples) and TGS (PB samples only). Copy number variation (CNV) analysis was performed for aneuploidy screening. Single-nucleotide polymorphisms (SNPs) from TE samples and parental peripheral blood were analyzed to determine the origin of CNVs and investigate discrepancies between TE- and PB-based PGT-A results. Clinical information from patients was collected for inter-group statistical comparisons.

**MAIN RESULTS AND THE ROLE OF CHANCE:**

The amplification success rate was 97.75% (87/89) for PB1 and 92.13% (82/89) for PB2, while the amplification success rate for PB1 + PB2 was 97.22% (35/36), comparable to that of TE-biopsied cells. The concordance rate of CNV results between NGS and TGS was 96.81%, with observed discrepancies primarily attributed to differences in the sizes of segmental imbalances and varying levels of intermediate copy numbers. However, when inferring oocyte ploidy status from PB analysis, the concordance rate with TE-biopsied CNV results (blastocyst formation rate 59.2%) was 75.68% (56/74). Among the 56 embryos with consistent results, the CNV profiles of 40 embryos were identical, while the remaining 16 were embryos with paternal meiotic or mitotic abnormalities. Our results demonstrated a higher euploidy rate with PB-based PGT-A (55.4%) compared to blastocyst-stage PGT-A (43.1%). Additionally, the euploidy rate in oocytes from AMA patients (>38 years) was 40%, which was lower than that in younger patients (≤38 years; 64%).

**LIMITATIONS, REASONS FOR CAUTION:**

PGT-A via PB biopsy is subject to specific technical limitations and clinical risks that warrant attention. PB biopsy for PGT-A can only detect maternal meiotic abnormalities; it cannot detect mitotic errors or paternal meiotic abnormalities. Moreover, PBs are single cells with limited DNA quantity and are prone to degradation over time. The timing and technical execution of the biopsy are critical for the success of WGA. Amplification failure can occur due to sample loss, experimental error, or the absence of genetic material in PBs resulting from meiotic errors.

**WIDER IMPLICATIONS OF THE FINDINGS:**

PB analysis represents a minimally invasive strategy that reduces the number of cryopreserved aneuploid embryos, decreases the number of embryo transfers required per live birth, and lowers miscarriage rates. Furthermore, TGS improves the cost-effectiveness and shortens the turnaround time of PB-based PGT-A, making it particularly suitable for fresh cleavage-stage (D3) transfers. This TGS-enhanced PB approach offers a clinically viable alternative to conventional TE biopsy by effectively combining the historical benefits of PB analysis with recent technological advances. The method shows significant promise for optimizing both clinical outcomes and laboratory efficiency in ART practice. Beyond PGT-A, the future may see the development of comprehensive, integrated PGT platforms (including PGT-M for monogenic disorders and PGT-SR for structural rearrangements) based on PB biopsy.

**STUDY FUNDING/COMPETING INTEREST(S):**

This work was supported by the National Natural Science Foundation of China (Grant No. 82273465) and the Pioneer and Leading Goose R&D Program of Zhejiang (Grant No. 2023C03034). All authors declare no competing interests.

**TRIAL REGISTRATION NUMBER:**

N/A.

WHAT DOES THIS MEAN FOR PATIENTS? This study explored a less invasive genetic testing approach for embryos during IVF, using genetic material from polar bodies (which are natural byproducts of egg development) instead of cells from the embryo itself. By combining this method with rapid modern sequencing technology (nanopore sequencing), we developed a faster and potentially safer way to identify chromosomally normal embryos for transfer.

Our results show that this polar body-based testing is a reliable option, especially for women of advanced maternal age. It can reduce the risk of miscarriage and the number of embryo transfers needed to achieve a successful pregnancy. The speed of this new process also allows for a ‘fresh’ embryo transfer on Day 3, avoiding the need to freeze all embryos and wait for results. While this method primarily detects chromosome abnormalities originating from the egg, it offers a significant clinical advantage for many patients seeking a less invasive and more efficient genetic testing option during their IVF treatment.

## Introduction

Preimplantation genetic testing for aneuploidy (PGT-A) represents a significant advancement in assisted reproductive technology (ART). This technique entails analyzing the chromosomal composition of embryos prior to uterine implantation to select euploid embryos for transfer. PGT-A is primarily indicated for advanced maternal age (AMA), unexplained recurrent miscarriage, and unexplained recurrent implantation failure (RIF; Tian *et al.*, 2024). These evidence-based indications, endorsed by international reproductive medicine societies such as ESHRE and ASRM, target populations at elevated risk for embryonic aneuploidy, where PGT-A demonstrates proven efficacy in enhancing live birth rates per transfer cycle while reducing miscarriage incidence through the selection of euploid embryos (Morales, 2024).

However, trophectoderm (TE)-based PGT-A faces increasing challenges. A primary challenge is embryonic mosaicism. Cellular heterogeneity within biopsy samples, amplification biases from minimal biopsy material, and systemic errors in sequencing and bioinformatic analysis frequently yield substantial rates of putative mosaic embryo diagnoses. Crucially, conventional PGT-A workflows with limited sequencing depth lack the discriminatory capacity to reliably distinguish true mosaicism from false mosaicism. False-positive mosaic diagnoses may result in the discard of 20–30% of embryos with developmental potential (Paulson, 2020; Treff and Marin, 2021). Conversely, embryos with confirmed true mosaicism retain documented reproductive competence, supported by multiple peer-reviewed reports of healthy live births following the transfer of mosaic embryos, underscoring the developmental plasticity of early human embryos (Barad *et al.*, 2022; Campos *et al.*, 2023). A second major concern is the invasive nature of TE biopsy, which may impair embryo implantation potential and adversely affect clinical outcomes (Schenk *et al.*, 2018; Rubino *et al.*, 2020). Invasive PGT-A faces regulatory restrictions or outright prohibition due to ethical concerns in certain countries. Furthermore, TE-based PGT-A demonstrates no statistically significant improvement in cumulative live birth rates (CLBRs) compared to conventional morphology-based embryo selection (Yan *et al.*, 2021). Although TE biopsy remains a valuable tool for detecting chromosomal abnormalities, its limitations in accurately identifying genetic mosaicism, its potential for embryo damage, and its inability to significantly enhance CLBRs must be carefully considered.

Polar body (PB)-based PGT-A represents a historically validated approach that effectively reduces miscarriage rates and improves live birth rates (Feichtinger *et al.*, 2015; Verpoest *et al.*, 2018), comparable to TE-based PGT-A. PB-based PGT-A offers several distinct advantages. First, since both the first (PB1) and second polar bodies (PB2) are single cells, PB-based PGT-A theoretically eliminates mosaic artifacts, thereby providing an unequivocal assessment of maternal meiotic errors (Hou *et al.*, 2013). Second, the minimally invasive nature of PB biopsy has a negligible impact on embryos (Strom *et al.*, 2000; Verdyck *et al.*, 2023). Furthermore, PB-based PGT-A permits cleavage-stage embryo (D3) transfer, circumventing the requirement for extended *in vitro* culture to the blastocyst stage (Oberle and Feichtinger, 2025). This substantially mitigates embryo attrition during prolonged culture, proving particularly advantageous for patients with limited embryo cohorts.

Nevertheless, PB-based PGT-A has been largely superseded by TE-based PGT-A in clinical practice due to inherent constraints. Its diagnostic scope is limited to maternally derived meiotic anomalies, failing to detect paternal meiotic errors or post-zygotic mitotic abnormalities. Technical challenges include rapid degradation of polar bodies, which require precise biopsy timing (∼9 h post-ICSI) (Montag *et al.*, 2013), suboptimal whole-genome amplification (WGA) success rates, and elevated costs relative to TE biopsy. These limitations explain the current restricted clinical implementation of PB-based PGT-A despite its unique benefits for selected patient populations.

The innovative convergence of nanopore sequencing, a third-generation sequencing (TGS) technology, with established PB biopsy methodologies holds significant potential to revitalize PGT-A. Nanopore sequencing demonstrates high accuracy and reliability in detecting chromosomal aneuploidy in PBs, with studies reporting up to 97.7% concordance with array-based comparative genomic hybridization (aCGH) (Madritsch *et al.*, 2024; Oberle *et al.*, 2024). Combining nanopore sequencing with PB biopsy enables rapid generation of PGT-A reports within a 9-h timeframe, facilitating fresh Day 3 embryo transfer and reducing the need for cryopreservation of aneuploid embryos (Verpoest *et al.*, 2018). Growing evidence suggests that the benefits of a ‘freeze-all’ strategy with elective frozen embryo transfer (FET) are not universal across all IVF populations. A large pragmatic randomized controlled trial (RCT) conducted by Wei *et al.* demonstrated that in women with a poor prognosis (defined by diminished ovarian reserve or low oocyte yield), a fresh transfer strategy resulted in a significantly higher live birth rate, clinical pregnancy rate and CLBR compared to a freeze-all strategy, challenging the assumption that deferred FET is universally beneficial (Wei *et al.*, 2025). Additionally, nanopore sequencers are cost-effective and flexible, making the technology accessible to a wider range of reproductive medicine centers. The streamlined experimental workflow reduces the technical burden on laboratory personnel. Flow cells permit sequential regeneration through washing protocols, enabling repeated reuse until nanopore functionality is exhausted. This distinctive feature eliminates the traditional requirement for batch processing of samples, facilitating truly on-demand, sample-to-answer genetic analysis (Tan *et al.*, 2023; Savva *et al.*, 2025).

In this study, polar bodies were collected from 125 oocytes obtained from 30 couples. Among these, 74 corresponding embryos developed into blastocysts, and TE biopsies were also performed. Both polar bodies and corresponding TE cells underwent WGA by multiple annealing and looping-based amplification cycles (MALBAC) (Zong *et al.*, 2012; Huang *et al.*, 2015). PB-derived DNA was subjected to both next-generation sequencing (NGS) and TGS, while TE biopsy samples were analyzed using NGS only. Copy number variations (CNVs) and single-nucleotide polymorphisms (SNPs) were obtained for aneuploidy screening and CNV origin analysis. Clinical information and peripheral blood samples were collected from the patients to enable inter-group statistical comparisons and to investigate discrepancies between TE-based and PB-based PGT-A results. Our findings demonstrate that TGS-enhanced PB analysis presents a clinically viable alternative to conventional TE biopsy, effectively integrating the historical benefits of PB analysis with contemporary technological advances.

## Materials and methods

### Clinical information of enrolled patients

In this comparative study, all patients undergoing PGT-A between October 2024 and May 2025 were enrolled. Samples that failed to meet predefined quality thresholds or in which analytical failure occurred were excluded from subsequent comparisons. Exclusion criteria included: (i) premature ovarian failure, (ii) polycystic ovary syndrome, and (iii) grade III endometriosis. Written informed consent was obtained from all participants following comprehensive counseling, and the study protocol was approved by the Reproductive Medicine Ethics Committee of the First Affiliated Hospital of Naval Medical University (approval no. CHRE-2024-06).

This study enrolled 30 couples with a comprehensive collection of clinical data and peripheral blood samples, comprising female patients presenting with RIF (n = 2), recurrent miscarriages (n = 7), AMA (>38 years old, n = 10), and adverse pregnancy histories (n = 3) alongside one male patient with severe teratozoospermia who required donor sperm fertilization, as detailed in Supplementary Table S1.

### ICSI and* in vitro* embryo culture

Briefly, for ICSI, transvaginal ultrasound-guided oocyte retrieval was performed 33–36 h after hCG administration. The cumulus–oocyte complexes (COCs) were then isolated from the follicular fluid, rinsed, and cultured in an incubator. After that, we proceeded with ICSI fertilization at 41–43 h after hCG administration. Fertilization was identified by the presence of two pronuclei.

### Polar body biopsy and blastocyst biopsy

One hour before polar body biopsy (PBB), the biopsy dish was prepared. Then, PB biopsy involved three different procedures. (1) The embryologist hatched the zona pellucida with a laser, and a simultaneous biopsy of both PB1 and PB2 was carried out at 18–19 h after ICSI, and then transferred to the same PCR tubes. (2) A biopsy of PB1 was carried out at 0.5 h after ICSI, and a biopsy of PB2 was carried out at 18–19 h after ICSI and then transferred to different PCR tubes. (3) Simultaneous biopsy of both PB1 and PB2 was carried out at 18–19 h after ICSI, then transferred to different PCR tubes.

### Single-cell whole-genome amplification

The obtained cells underwent WGA and NGS library preparation using the ChromInst™ Universal Library Preparation Kit (Yikon Genomics, Suzhou, China) following the provided operational guidelines. Briefly, after cellular lysis, the liberated genomic DNA was primed with a random primer library, initiating pre-amplification, which was followed by exponential amplification to achieve an output of 150 ng for TE cells or 15 ng for PB cells. Agarose gels (1.5%) were used to assess the size of the DNA fragments. Detailed experimental processes of WGA can be found in Supplementary File S1 and in Supplementary Tables S2, S3, S4, S5, and S6.

### Library preparation and nanopore sequencing

For the preparation of Oxford Nanopore Technologies libraries, 10 ng of amplified DNA was used per sample. The DNA fragment ends were repaired using the Ultra II End Repair/dA-tailing module (New England Biolabs, Ipswich, MA, USA). Subsequently, 1.25 µl of barcode adapter and 5 µl of Blunt/TA Ligase Master Mix (New England Biolabs) were added to each reaction. The mixture was thoroughly homogenized using a multichannel pipette and incubated at 25°C for 10 min in a thermocycler. The adapter provided in the NBD114.24 kit (Oxford Nanopore Technologies, Oxford, UK) was used for subsequent ligation steps. Quantification of the library fragments was performed using a Qubit^®^ 3.0 fluorometer (Thermo Fisher Scientific, Waltham, MA, USA). Subsequently, the libraries were sequenced using the P2 Solo sequencer (Oxford Nanopore Technologies) and its corresponding R10.4.1 flow cells (Oxford Nanopore Technologies), from which the data were obtained. Sequencing was performed strictly in accordance with the manufacturer’s protocol. Each sample was sequenced with about 1.5M reads (about 0.1×).

### Copy number variation analysis based on NGS and nanopore sequencing

For TGS data, raw electrical signals (POD5 format) were basecalled into nucleotide sequences using Dorado (Oxford Nanopore Technologies, v0.9.1) and converted to FASTQ format. The resulting reads were processed with fastp (v0.26.0) (Chen *et al.*, 2018) to trim 150 bases from the 5′-end and 50 bases from the 3′-end of each read. Reads containing any base with a Phred quality score below 7 were discarded, and only reads with a minimum length of 200 bases after trimming were retained for downstream alignment. For NGS data, raw sequencing reads were quality-filtered using fastp (v0.26.0) with adapter trimming and quality control. The remaining bioinformatics analyses were the same for TGS data and NGS data. Processed reads were aligned to the human reference genome (GRCh37/hg19) using bwa-mem2 (v2.2.1) (Li and Durbin, 2009). Duplicate reads were marked and removed via Sambamba (v0.8.0) (Tarasov *et al.*, 2015).

The genome was partitioned into 400 kb sliding windows with a 200 kb step size (yielding approximately 30 000–35 000 bins), excluding satellite regions, centromeres, telomeres, and other highly repetitive genomic segments with inherently poor mappability. Read counts per window were normalized for GC bias using locally weighted scatterplot smoothing (LOESS) regression, which modeled the relationship between GC content and read depth to compute corrected counts. These normalized values were then compared to a reference set of control samples to calculate relative copy number ratios and *Z*-scores. Sex chromosome corrections were applied based on the sample’s genetic sex.

Copy number ratios were calculated and segmented using the Circular Binary Segmentation (CBS) algorithm (DNAcopy package in R) (Lucito *et al*., 2003), with aberrant segments (log_2_ ratio ± 0.25) automatically identified as putative CNVs. The reporting criteria included whole-chromosome-level abnormalities, whole-arm level abnormalities, and segmental abnormalities no smaller than 4 Mb (at least 20 points to support with a 200 kb step size). For mosaic abnormalities, the thresholds were set at a segment size of at least 10 Mb and a mosaicism level exceeding 30%. The whole process of CNV detection is shown in Supplementary Fig. S1.

Four QC terms were used, including MAPD (median absolute pairwise difference, should ≤0.25), BinCV (CV of all bins on the genome, should ≤0.2), CNVMergeCV (CV of all segments, should ≤0.2), and CNVpqCV (CV of arms of all chromosomes, should ≤0.15). When MAPD/BinCV and CNVMergeCV/CNVpqCV were too high, the sample QC would fail.

### Heteroploidy detection and CNV origin analysis

The initial processing of raw sequencing data involved quality assessment and adapter removal using fastp (v0.26.0). Cleaned reads were aligned against the human reference genome (hg19) with bwa-mem2 (v2.2.1) to produce BAM format alignments. Read duplicates were identified and eliminated employing Samblaster (v0.1.26) (Faust and Hall, 2014). Quality metrics such as read depth, variant allele frequency (VAF), base quality, and genotype quality were collected from the BAM files using samtools (v1.16) (Danecek *et al.*, 2021) and GATK (v4.2.0) (McKenna *et al.*, 2010). These metrics were aggregated and summarized via MultiQC (v1.14) (Ewels *et al.*, 2016). Finally, Bcftools (v1.16) (Danecek *et al.*, 2021) was used for variant calling and filtering, resulting in VCF files.

To identify embryonic heteroploidy, cluster profiles were constructed based on the VAF of SNPs with at least 20× read coverage. For every embryo, biallelic SNPs from autosomes were subjected to density-based clustering implemented in hdbscan (v0.8.27) (McInnes *et al.*, 2017). The parameter min_cluster_size was optimized by maximizing the silhouette coefficient. In euploid embryos, VAF values typically form three clusters near 0, 0.5, and 1. Triploid samples generally showed four clusters centered around 0, 0.33, 0.67, and 1, while uniparental disomy (UPD) was characterized by two clusters at 0 and 1.

To determine the parental origin of CNVs, an uneven score was calculated as a statistical indicator of allelic imbalance. This score was derived from VAF values of informative SNPs exhibiting paternal AA and maternal BB genotypes, or vice versa. The uneven score was computed as log_2_(N_mat_/N_pat_), where N_mat_ and N_pat_ represent the summed counts of maternal- and paternal-biased loci per chromosome, respectively. A value of 1 indicates no parental bias. A reference distribution of uneven scores was established using control samples, following a positively skewed normal distribution. Chromosomal segments were classified as exhibiting maternal or paternal bias if their uneven scores exceeded the upper threshold (mean + 2 × SD) or fell below the lower threshold (mean − 2 × SD), with the mean and SD derived from the reference population.

## Results

### WGA results of PB and TE biopsy cells

**Figure 1.**
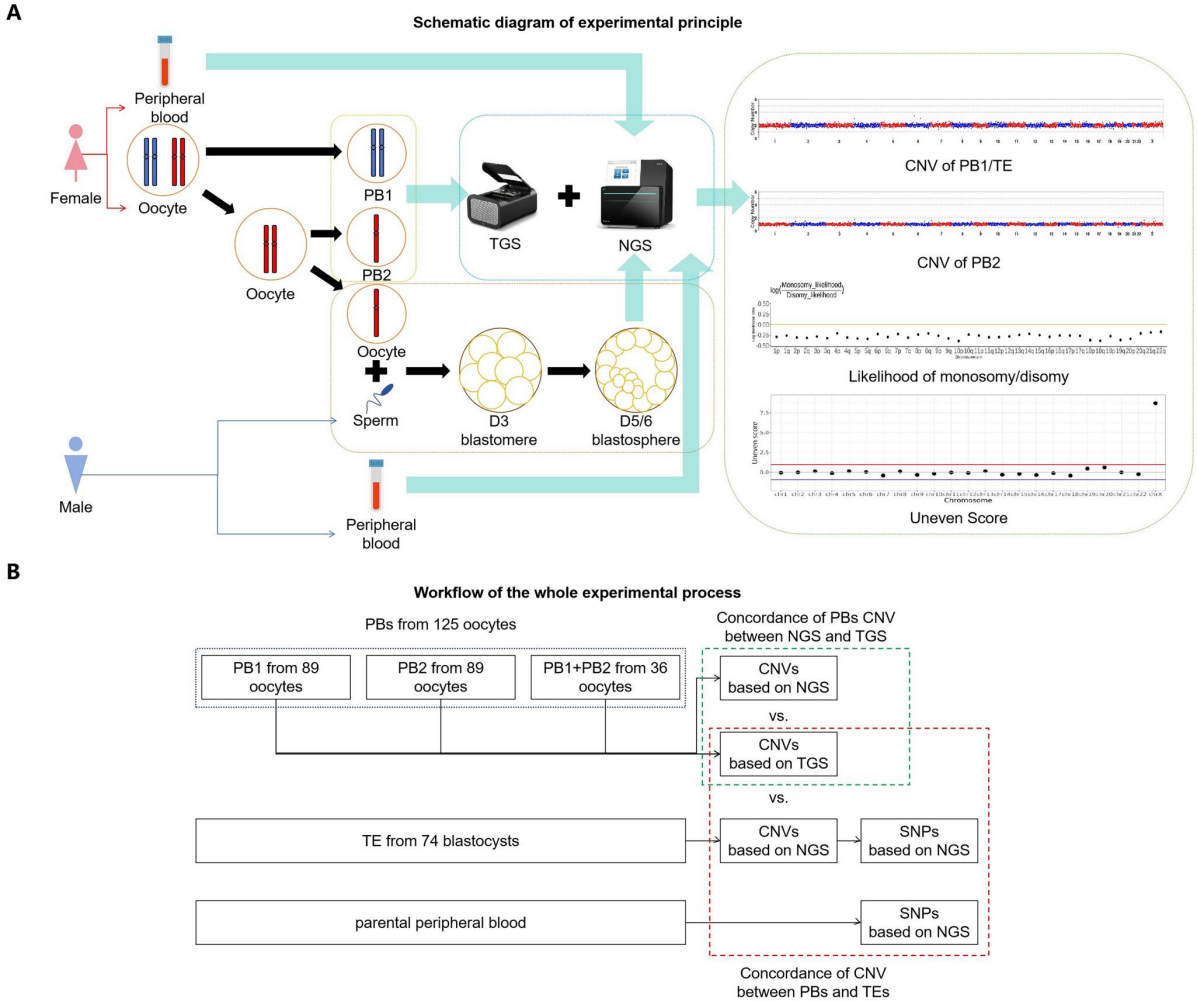
**Technical workflow schematic.** (**A**) Schematic diagram of experimental principle. Peripheral blood samples, oocytes, and sperm were collected from both female and male partners. Following ICSI, a polar body (PB) biopsy was performed. The resulting zygotes were cultured to the blastocyst stage (D5/6), followed by trophectoderm (TE) biopsy. All PB and TE samples underwent single-cell whole-genome amplification (WGA) via multiple annealing and looping-based amplification cycles (MALBAC), followed by library preparation. PB-derived DNA was subjected to both next-generation sequencing (NGS) and third-generation sequencing (TGS), while TE biopsy samples were analyzed exclusively by NGS. Sequencing data were processed to generate copy number variation (CNV) profiles, euploidy assessments, and origin-tracing analyses of chromosomal abnormalities integrated with parental single-nucleotide polymorphism (SNP) profiles obtained from peripheral blood samples. (**B**) Workflow of the whole experimental process. TGS-based CNV results and NGS-based CNV results were compared. In addition, the CNV results of PBs and corresponding TEs were compared. In the case of inconsistent results, the authenticity and origin of CNV abnormalities were analyzed through SNPs by sequencing the peripheral blood samples of parents.

We analyzed paired polar bodies from 125 oocytes, including 89 individually amplified and sequenced first polar bodies (PB1) and second polar bodies (PB2) and 36 jointly processed PB1 + PB2 samples. From the 74 corresponding fertilized oocytes, which successfully developed into blastocysts (59.2% blastulation rate), TE biopsies were obtained. Both the polar bodies and the corresponding TE cells underwent WGA using MALBAC. The amplified products from the polar bodies were sequenced using both NGS and TGS, while the amplified products from the TE-biopsied cells and gDNA from peripheral blood were sequenced only using NGS. CNVs and SNPs were obtained for aneuploidy screening and CNV origin testing. CNV origin analysis through parental blood SNP haplotyping was performed to investigate diagnostic discrepancies between TE-based PGT-A and PB-based PGT-A results. Clinically stratified subgroup comparisons were realized based on patient phenotypes. The technical scheme diagram of this study is shown in Fig. 1.

**Figure 2.**
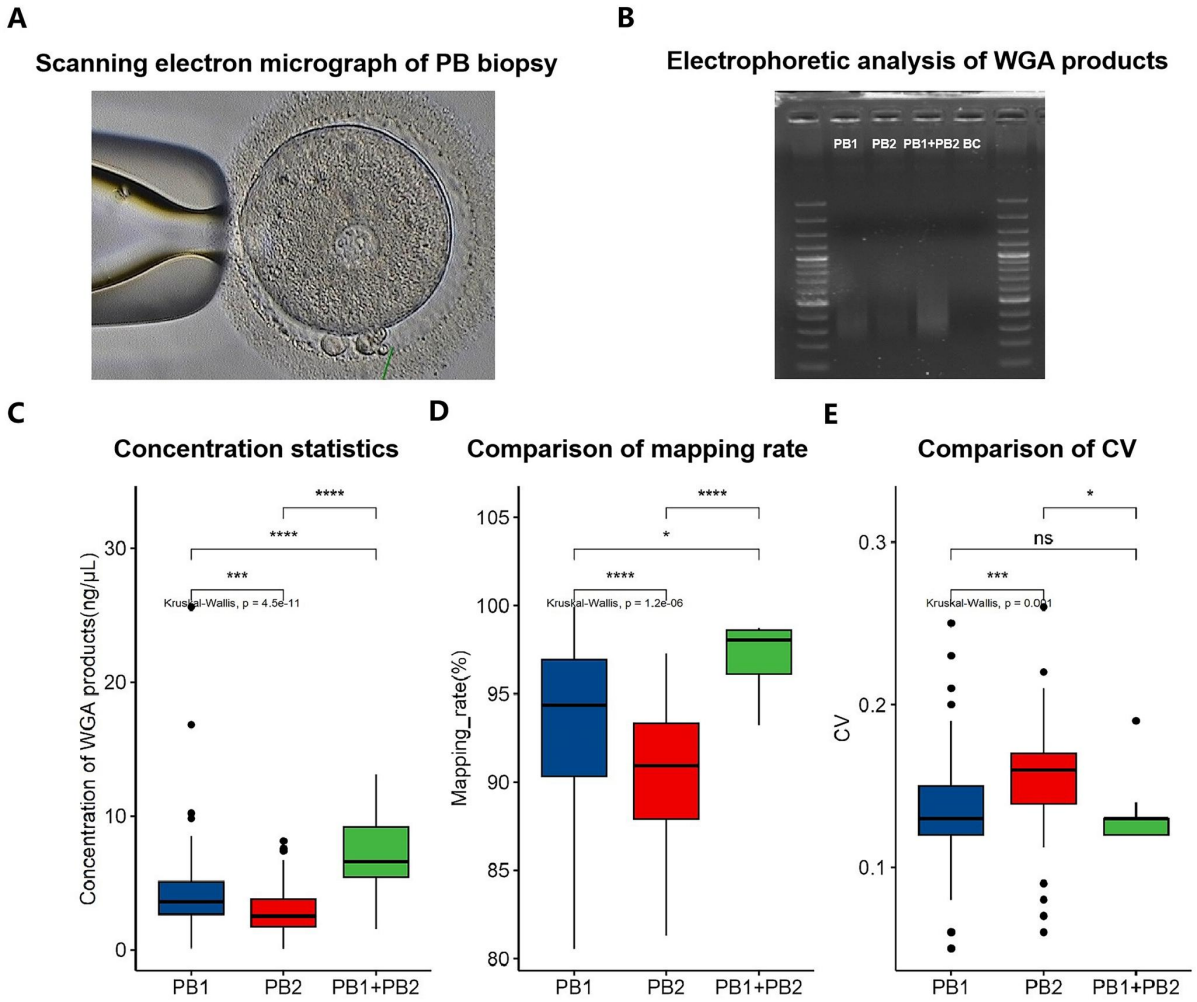
**Quality control analyses.** (**A**) Scanning electron micrograph of polar body (PB) biopsy; (**B**) electrophoretic analysis of PB whole-genome amplification (WGA) products, including the first polar body (PB1), the second polar body (PB2), PB1 + PB2, and blank control (BC); (**C**) quantitative distribution of PB amplification product concentrations; (**D**) statistical distribution of mapping rate for PBs; (**E**) statistical distribution of coefficient of variation (CV) for PBs.

Single-cell WGA was performed using MALBAC for PB1, PB2, PB1 + PB2, and corresponding TE-biopsied cells. The amplification success rates were 97.75% (87/89) for PB1, 92.13% (82/89) for PB2, 97.22% (35/36) for PB1 + PB2, and 100% (74/74) for TE-biopsied cells. The relatively low amplification success rate of PB2 may be attributed to its haploidy, containing only half the DNA content of PB1 and TE cells. Additionally, both the timing and technical execution of PB biopsy significantly impacted amplification success rates (Montag *et al.*, 2013). Figure 2A illustrates a PB biopsy photo post-ICSI.

Electrophoretic analysis (Fig. 2B) demonstrated the expected molecular weight distribution of amplification products, confirming successful WGA. As shown in Fig. 2C, the concentration distribution patterns revealed significant differences across different sample types (*P* < 0.01). The amplification concentration of PB1 + PB2 is greater than that of PB1, and the amplification concentration of PB1 is greater than that of PB2. The concentrations of amplified products were quantified, with detailed results are presented in Supplementary Table S7.

### Validation of nanopore sequencing for CNV detection of PBs

We first performed a quality control (QC) assessment of the sequencing data from PBs and TE samples. Key QC parameters are also affected by the concentration of amplification products, including the mapping rate and coefficient of variation (CV), among others. The distributions of the mapping rate and CV for PBs are presented in Fig. 2D and E. The QC pass rates were as follows: PB1 (95.40%, 83/87), PB2 (85.37%, 70/82), PB1 + PB2 (97.14%, 34/35), and TE-biopsied cells (100%, 74/74).

**Figure 3.**
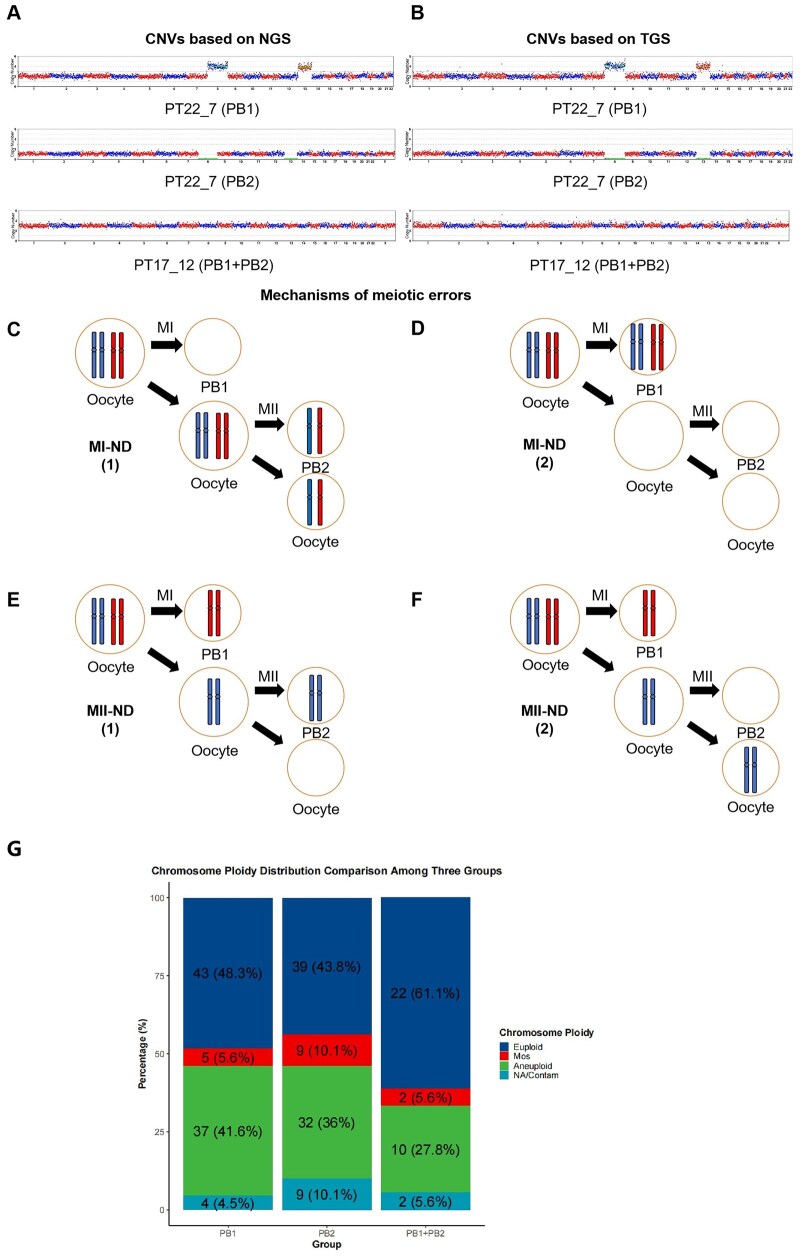
**Polar body (PB) copy number variation (CNV) profiles and mechanisms of meiotic errors.** (**A**) Representative next-generation sequencing (NGS) and (**B**) third-generation sequencing (TGS)-based CNV results from the first polar body (PB1), the second polar body (PB2), and combined PB1 + PB2 analyses; Schematic illustration of meiotic error mechanisms, including (**C**, **D**) meiosis I non-disjunction (MI-ND) and (**E**, **F**) meiosis II non-disjunction (MII-ND), highlighting chromosomal segregation defects during oogenesis; (**G**) comparison of euploidy, aneuploidy and mosaicism (intermediate CNVs) rates among PB1, PB2, and PB1 + PB2.

To assess the reliability of nanopore sequencing, we performed parallel NGS and nanopore sequencing on PBs. Figure 3A shows the CNV graphs based on NGS, while Fig. 3B shows the CNV graphs based on TGS for the same samples. The gain or loss of CNV in PBs comes from abnormalities during the meiosis of oocytes. Figure 3C–F shows some possible mechanisms of CNV abnormality, including meiosis I non-disjunction (MI-ND), meiosis II non-disjunction (MII-ND), and precocious separation of sister chromatids (PSSC) (Verdyck *et al.*, 2023). Comparative analysis of CNV results revealed a 96.81% concordance rate between NGS and nanopore sequencing. Discrepancies primarily arose from variations in segmental imbalance sizes and levels of intermediate copy number. Notably, the overall trends of CNV signals were completely consistent between NGS and nanopore sequencing. When minor differences in segmental size and intermediate copy number level were disregarded, the concordance rate reached 100%, demonstrating the high methodological reliability of nanopore sequencing.

Unexpected intermediate copy numbers were detected in 7.48% of PB1, PB2, and PB1 + PB2 samples. Mosaicism should theoretically be absent in single-cell PBs, which likely represent technical artifacts arising from single-cell amplification and PB degradation. We attempted to establish a PB-specific reference system to mitigate these artifacts but observed only marginal improvement. Furthermore, 1.87% (4/214) of PB samples (two PB1 and two PB2) exhibited a low proportion of Y chromosome signals in their CNV profiles, which are biologically unexpected given the maternal origin of PBs. These faint Y chromosome signals originate from exogenous contamination introduced during the biopsy procedure or WGA. Consequently, the presence of Y chromosome signals serves as a quality control indicator for potential sample contamination. A high proportion of Y chromosome signals (higher than 10%) suggests that the CNV results of the corresponding PBs are unreliable and should be interpreted with caution. Complete CNV profiles for all PBs are provided in Supplementary Table S8.

Indeed, two distinct biopsy methods were employed in this study: one involving separate biopsy, amplification, and sequencing of PB1 and PB2, and the other utilizing simultaneous biopsy and co-amplification of PB1 and PB2. Interestingly, Fig. 3G shows that the PB1 + PB2 co-amplification approach yielded the highest euploidy rate (61.11%), along with the lowest rates of mosaicism (5.56%) and aneuploidy (27.78%). These findings suggest that both the initial quantity of nucleic acid template and the timing of biopsy can significantly influence the CNV results of PBs. For clinical applications, we recommend the simultaneous biopsy and co-amplification of PB1 and PB2, as this method not only simplifies operational procedures but also generates more reliable CNV outcomes.

### Exploration of abnormal results based on SNPs

Whole-genome low-pass sequencing-derived SNPs enabled concurrent assessment of PB chromosomal ploidy status and contamination detection through integrated analysis with WGA product concentrations, thereby identifying sample mix-ups (e.g. PB1/PB2 misidentification) and ensuring diagnostic reliability (McCoy *et al.*, 2023; Tikhonov *et al.*, 2024). Consistent with biological expectations, PB1 exhibited diploid genomic content, PB2 demonstrated haploid profiles, while combined PB1 + PB2 analyses yielded triploid signatures.

**Figure 4.**
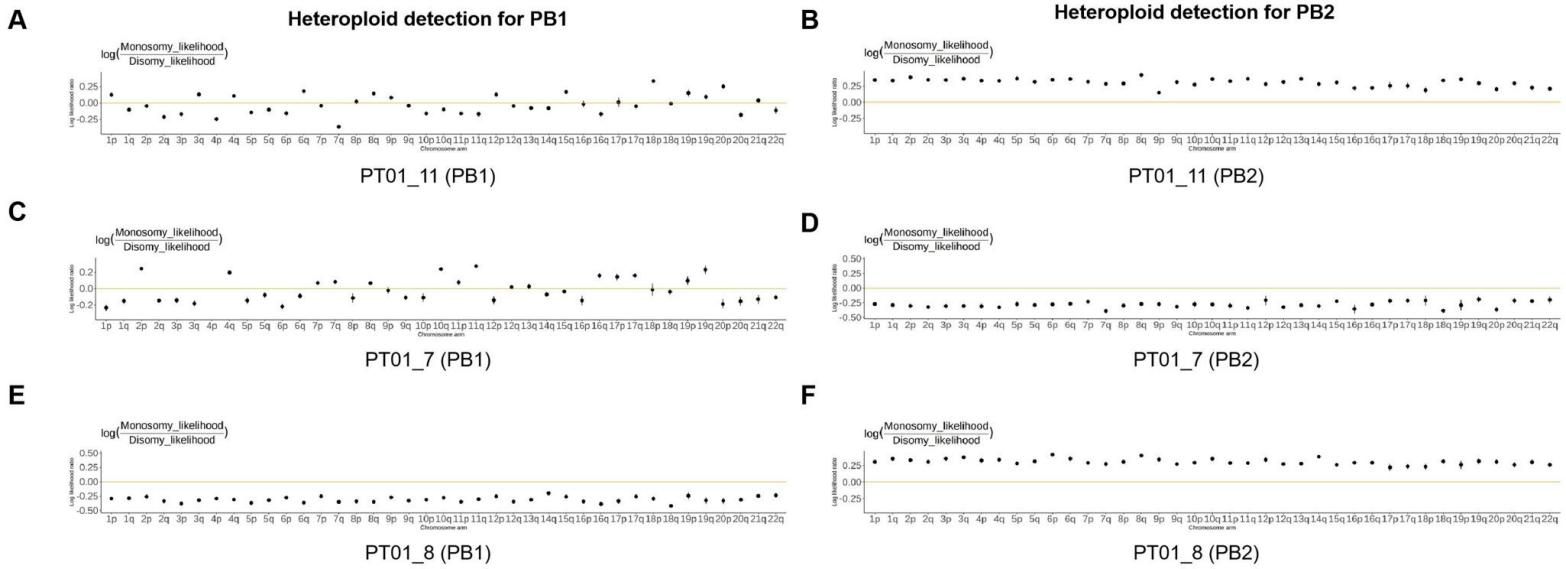
**Abnormal sample monitoring.** Detection of contamination in polar bodies (PBs) based on heteroploid analysis. The horizontal axis represents individual chromosomes, while the vertical axis indicates the likelihood ratio. Data points (black dots) located above the yellow horizontal line suggest a tendency toward haploidy/monosomy for the corresponding chromosome, whereas points below the yellow line indicate a tendency toward diploidy/disomy. (**A**) PT01_11 (PB1) displayed normal diploid characteristics with fluctuating variant allele frequency (VAF); (**B**) PT01_11 (PB2) exhibited haploid patterns; (**C**) PT01_7 (PB1) presented standard diploid features; (**D**) PT01_7 (PB2) demonstrated anomalous diploidization suggestive of granulosa cell contamination; (**E**) PT01_8 (PB1) showed diploid signatures. (**F**) PT01_8 (PB2) showed normal haploid..

Representative VAF analyses from family PT01 exemplify this quality control framework. PT01_11 (PB1) displayed normal diploid characteristics with fluctuating VAF values indicative of homologous recombination events (Fig. 4A), whereas PT01_11 (PB2) exhibited definitive haploid patterns (Fig. 4B). Notably, while PT01_7 (PB1) presented standard diploid features (Fig. 4C), PT01_7 (PB2) demonstrated anomalous diploidization suggestive of granulosa cell contamination (Fig. 4D). PT01_8 (PB1) showed aberrant diploid signatures with elevated WGA product concentrations consistent with multi-cell contamination (Fig. 4E). Figure 4F shows normal haploid PT01_8 (PB2).

### Concordance analysis between oocyte and blastocyst CNV profiles

For embryos where PB1 and PB2 were analyzed separately, oocyte CNV profiles were reconstructed through computational simulation of meiotic segregation patterns. In contrast, when both PB1 and PB2 were simultaneously biopsied and sequenced, the corresponding oocyte CNV status could be determined through direct genomic deduction.

**Figure 5.**
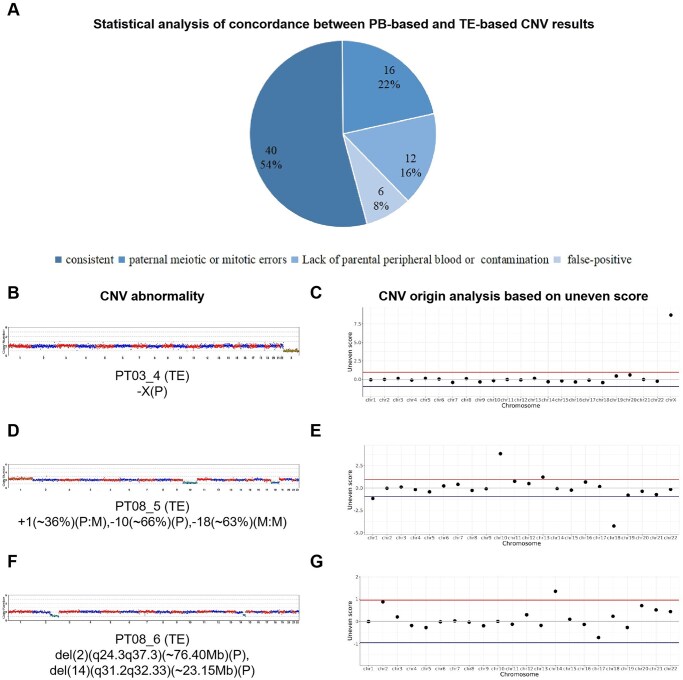
**Analysis of the inconsistency of copy number variations (CNVs) between oocytes and blastocysts.** (**A**) Statistical analysis of concordance between polar body (PB)-based and trophectoderm (TE)-based CNV results. (**B)** shows the whole-chromosome-level CNV abnormalities of TE-based preimplantation genetic testing for aneuploidy (PGT-A), while corresponding PB-based PGT-A shows euploidy. (**C)** shows the uneven score based on single-nucleotide polymorphisms (SNPs). The SNP information indicates that the X chromosome is more inclined to come from the maternal (M) source, which means that the absence of the X chromosome from the paternal (P) source causes the SNP on the X chromosome to deviate from the paternal source; (**D**, **E**) mosaic-level inconsistency; (**F**, **G**) segmental-level inconsistency.

Following the derivation of oocyte CNV profiles, we compared oocyte CNV results with corresponding TE-based CNV results. All oocyte CNV results inferred from PBs based on TGS, as well as corresponding blastocyst CNV results based on NGS, are listed in Supplementary Table S9. In this table, we only present the paired PBs and TEs CNV results for comparison of their differences. ‘P’ represents that the abnormal CNV originates from paternal side, and ‘M:M’ represents that the abnormal CNV originates from maternal side and is from mitosis, while ‘Balanced’ indicates that the aneuploid or mosaic abnormalities of TEs are false, and the CNV abnormality does not truly exist because the SNP allele frequencies from the maternal and paternal sources are equal and balanced. The results from Supplementary Table S9 were subjected to categorized statistical analysis and are visually summarized in Fig. 5A. Among 74 paired analyses, 40 cases demonstrated complete concordance. For the 34 discordant cases, we performed additional sequencing of parental peripheral blood samples to obtain uneven scores (refer to Methods section) to determine the origin of blastocyst CNV abnormalities (Taylor *et al.*, 2014; Harton *et al.*, 2017; Vera-Rodriguez and Rubio, 2017). There was a lack of parental peripheral blood samples, amplification failure, or contamination in PBs in 12 cases, making it impossible to determine the origin of the discrepancy. Uneven scores revealed that 16 cases resulted from paternal meiotic or mitotic errors, while 6 cases represented false-positive calls in PBs CNV analysis. Thus, the concordance rate with TE-biopsied CNV results was 75.68% (56/74). No false-negative cases (undetected maternal meiotic errors) were observed. As illustrated in Fig. 5B–G, the discrepant cases were systematically analyzed based on uneven score (refer to Materials and methods section).

To validate blastocyst PGT-A accuracy in cases of PBs false-positives, we performed additional analysis of discarded embryos. These validation studies confirmed the accuracy of blastocyst PGT-A results and the occurrence of false-positive CNV calls in PBs analysis. Moreover, we found that almost all false-positive CNV abnormalities that appeared in PBs but did not appear in TEs were intermediate CNVs. We attribute these false-positives CNVs of PBs to either technical artifacts from single-cell amplification/degradation or potential embryonic self-correction during early development (Madritsch *et al.*, 2024).

### Statistical analysis of polar body PGT-A results and patient clinical information

**Figure 6.**
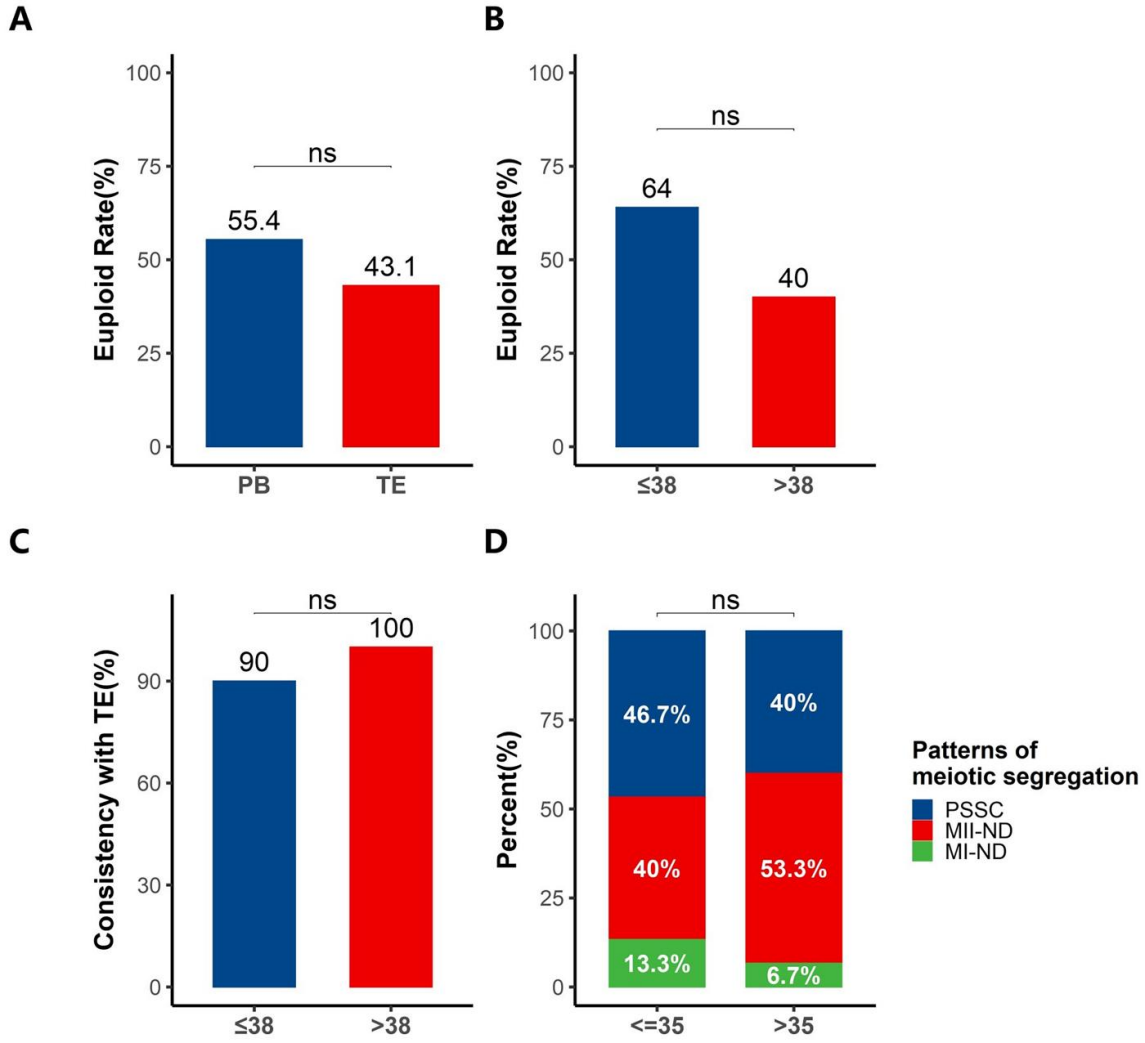
**Stratified statistical analyses of embryological and genetic outcomes.** (**A**) Euploidy rates between polar body (PB)- and trophectoderm (TE)-based preimplantation genetic testing for aneuploidy (PGT-A). (**B**) Age-stratified euploidy rates in younger (≤38 years) versus advanced maternal age (AMA; >38 years) cohorts. (**C**) Concordance rates between PB and TE-based PGT-A results stratified by maternal age. (**D**) Distribution of meiotic error origins, i.e. meiosis I non-disjunction (MI-ND) versus meiosis II non-disjunction (MII-ND) in younger versus AMA patients. ns, no significance.

The overall blastocyst formation rate was 59.2% (74/125). Notably, AMA patients (>38 years) exhibited reduced blastocyst formation (38.89% vs. 72.5% in younger patients). This demonstrates that *in vitro* culture leads to substantial oocyte attrition, especially in AMA patients. The higher euploidy rate in PB-based PGT-A (55.4%) compared to TE-based PGT-A (43.1%) suggests that paternal meiotic/mitotic errors impact blastocyst chromosomal status (*P* = 0.169, Fig. 6A). Pre-D3 PGT-A with cleavage-stage transfer could optimize oocyte utilization in AMA patients by avoiding culture-associated embryo loss and preserving mosaic embryos compromised by mitotic errors.

Our analysis also revealed age-related differences in both euploidy rates and diagnostic concordance. For AMA patients (>38 years), the euploidy rate was 40%. While for younger patients (<38 years), the euploidy rate was 64% (*P* = 0.0625, Fig. 6B). As for PB–TE concordance, the consistency rate in younger patients was 90%, while in AMA patients, it was 100% (*P* = 0.562, Fig. 6C). The higher PB–TE concordance in AMA patients suggests an increased predominance of maternal meiotic errors (detectable by PB analysis), whereas paternal contributions (paternal meiotic/mitotic errors) may disproportionately affect younger cohorts.

In addition, our analysis revealed distinct patterns of meiotic segregation errors between groups. The MII-ND incidence rate for AMA patients was 53.3%, which was higher than for younger patients (40%), indicating that MII-derived errors are dominant in the AMA cohort. There was a higher prevalence of MI-derived errors in younger cohorts (*P* = 0.706, Fig. 6D).

## Discussion

A total of 125 oocytes were collected from 30 couples. PB1 and PB2 from 89 oocytes were individually amplified and sequenced, while those from the remaining 36 oocytes were processed jointly (PB1 + PB2). The amplification success rate was 97.75% (87/89) for PB1 and 92.13% (82/89) for PB2, while the amplification success rate for PB1 + PB2 was 97.22% (35/36), comparable to that of TE-biopsied cells. The concordance rate of CNV results between NGS and TGS was 96.81%, with observed discrepancies primarily attributed to sizes of segmental imbalance and varying levels of intermediate CNVs. However, when inferring oocyte CNV from PB CNV, the concordance rate with TE-biopsied CNV results (with a blastocyst formation rate of 59.2%) was 75.68% (56/74). Among 56 embryos with consistent results, the CNVs of 40 embryos were exactly the same, while 16 embryos carried paternal meiotic or mitotic abnormalities. Our results demonstrated a higher euploidy rate in PB PGT-A (55.4%) compared to blastocyst-stage PGT-A (43.1%). Additionally, the euploidy rate in oocytes from AMA patients (>38 years) was 40%, which was lower than that in younger patients (≤38 years; 64%).

In this study, we compared the CNV results of PBs based on NGS with those based on nanopore sequencing and found a high degree of consistency (96.81%), while the inconsistent results were mainly attributed to minor differences in the levels of intermediate CNVs and the sizes of the segmental abnormalities. The consistency comparison demonstrated the accuracy of nanopore sequencing in detecting CNVs in PBs (Madritsch *et al.*, 2024; Oberle *et al.*, 2024). Additionally, we compared the consistency of CNVs between PB biopsy and blastocyst biopsy. For the inconsistent results, we conducted an analysis of the sources of copy number abnormalities and found that the inconsistencies were mainly caused by paternal meiotic and mitotic events. The consistency comparison with blastocyst PGT-A results validated that PGT-A based on PBs can accurately detect maternal meiotic abnormalities. By further categorizing the population of our study, we clearly defined the indications for PGT-A based on PBs.

We have developed an algorithm that can predict the CNVs of oocytes from the mixed sequencing data of the PB1 and PB2. Additionally, it is also possible to co-amplify and sequence the PB1 and PB2 together for CNV analysis, thereby directly obtaining the CNV results of the corresponding oocytes. When the PB1 and PB2 are amplified and sequenced separately, the CNV and SNP results of the polar bodies can be analyzed to determine whether they meet the expected outcomes and to identify any potential sample contamination or mix-up, which can serve as quality control indicators. However, for real-world clinical applications, we recommend co-biopsy and co-amplification sequencing of the PB1 and PB2. This approach simplifies the biopsy procedure, increases the amplified success rate, and reduces the mosaic rate of CNV results. Of course, the trade-off is that when the PB1 and PB2 are tested together, it is impossible to distinguish segregation errors between meiotic I and meiotic II, and the specific types and details of meiotic abnormalities cannot be clearly identified. When using polar bodies for PGT-M or PGT-SR, the PB1 and PB2 still need to be tested separately to confirm whether the oocyte carries the pathogenic mutation through linkage analysis (Wang *et al.*, 2021).

The rapid development of TGS has breathed new life into the traditional method of PB biopsy, opening up new application scenarios. Combining the flexible and portable nanopore sequencer with PB biopsy enables minimally invasive and rapid PGT-A testing within 6 h (Tan *et al.*, 2023). In our work, we have included two cases of rapid PGT-A testing in real clinical settings (PT10 and PT11). The procedure is as follows: (1) oocyte retrieval and ICSI on D0; (2) PB biopsy on the morning of D1; (3) WGA (2.5 h) and library preparation (1.5 h) on the afternoon of D1; (4) sequencing starting on the evening of D1 and running overnight; (5) bioinformatics analysis of the sequencing data to obtain PGT-A reports (0.5 h) on the morning of D2; (6) and fresh cleavage-stage embryo transfer on D3. One family (PT10) with an AMA woman transferred a euploid cleavage-stage embryo and achieved a successful ongoing pregnancy (Supplementary Fig. S2). However, several studies underscore the principle of individualized treatment, indicating that freeze-all is a powerful tool primarily for mitigating ovarian hyperstimulation syndrome (OHSS) risk in high-response patients but may not be advantageous, and could even be detrimental, for patients with a low ovarian response. Time to pregnancy can be assumed to be shorter using a fresh embryo transfer strategy in the case of similar CLBR, as embryo transfer is delayed in a freeze-all strategy (Wong *et al.*, 2017; Wei *et al.*, 2025). In addition, aneuploid embryos can be discarded, saving costs for subsequent culture and vitrification. For AMA women, low blastocyst formation rates and few euploid embryos are challenges (L'Heveder *et al.*, 2021). Use of the PB biopsy and TGS PGT-A protocol can increase the number of available embryos, providing clinical benefits to patients.

However, PGT-A via PB biopsy presents specific technical limitations and clinical risks that warrant attention. PB biopsy for PGT-A can only detect maternal meiotic abnormalities, not mitotic abnormalities or paternal meiotic abnormalities. Although most chromosomal abnormalities in embryos are of maternal origin, the indications for PB biopsy PGT-A must be carefully considered. Several studies (Capalbo *et al.*, 2013; Christopikou *et al.*, 2013; Chen *et al.*, 2025) have examined the accuracy and reliability of PB-based PGT-A by comparing CNV profiles derived from PBs with those from TE cells or blastomeres. Although limited in sample size, these investigations provide compelling evidence supporting the utility of PB biopsy as a valuable PGT-A strategy, while also clearly delineating its technical limitations. Collectively, these studies demonstrate that while PB biopsy is a highly accurate method for identifying meiotic-origin aneuploidies (100% concordant) and single-gene disorders of maternal inheritance, its clinical predictive value is ultimately constrained by post-zygotic events. Only 62 out of 78 (79.5%) of the abnormal meiotic segregations in the PBs were consistent with the aneuploidies observed in their corresponding TEs. And as high as 20% of female-derived aneuploidies detected on PBs were rescued at the blastocyst stage. The findings of these studies are basically consistent with our research results. The technique shows exceptional concordance for predicting cleavage-stage embryo status, and its ability to select euploid embryos for transfer without the need for extended culture offers a distinct advantage for patients with a limited ovarian reserve. However, the significant incidence of false-positives coupled with the biological phenomenon of aneuploidy rescue (e.g. trisomic diploidization) between the cleavage and blastocyst stages, underscores a critical limitation. Thus, the choice of biopsy stage should be a stratified decision, with PB analysis being a valuable option specifically for women with AMA and a low oocyte yield.

Additionally, PBs degrade over time, which can affect the accuracy of results (Oberle and Feichtinger, 2025). The timing and handling during biopsy are crucial. The optimal time for PB biopsy is within 9–14 h after ICSI. Improper biopsy timing or handling may accelerate DNA degradation, leading to false intermediate CNVs. Both NGS and TGS methods have reported intermediate CNVs, which are difficult to eliminate in bioinformatics analysis. It is recommended to use SNP information to assist in determining the authenticity of intermediate CNVs. Moreover, polar bodies are single cells with limited nucleic acid content, and WGA of a single cell carries a high risk of failure. The causes of single-cell amplification failure are diverse, including sample loss, experimental errors or the absence of genetic material in the PB due to meiotic errors. To minimize this risk, it is advisable to visually confirm the placement of the PB cells into the sampling tube under a microscope.

In TE-based PGT-A, the presence of mosaicism can lead to false-positive results, which may result in the abandonment of healthy embryos and a decrease in CLBRs (Madero *et al.*, 2023; Viville and Aboulghar, 2025). In contrast, PB analysis is a minimally invasive approach that reduces the number of cryopreserved aneuploid embryos, decreases the required embryo transfers per live birth, and lowers miscarriage rates. Furthermore, TGS enhances the cost-efficiency and shortens the reporting period of PB-based PGT-A, making it particularly suitable for fresh D3 blastomere transfers. This TGS-enhanced PB approach presents a clinically viable alternative to conventional TE biopsy, effectively combining the historical advantages of PB analysis with modern technological advancements. The method demonstrates particular promise for optimizing both clinical outcomes and laboratory efficiency in ART practice. In addition to the PGT-A based on PBs, comprehensive and integrated PGT technologies (including PGT-A, PGT-M, and PGT-SR) based on PB biopsy are anticipated in the future (Liu *et al.*, 2021; Peng *et al.*, 2023; Xia *et al.*, 2024). The TGS-enhanced PB approaches present clinically viable alternatives to conventional TE biopsy, effectively combining the historical advantages of PB analysis with modern technological advancements.

## Supplementary Material

hoaf069_Supplementary_Data

## Data Availability

The data are available from the corresponding author upon request.
