# Supplementary material for: Rapid and minimally invasive preimplantation genetic testing for aneuploidies (PGT-A) based on polar body and nanopore sequencing: a viable alternative to conventional trophectoderm-based PGT-A?
Source: Hum Reprod Open. 2025 Oct 30;2025(4):hoaf069. doi: 10.1093/hropen/hoaf069 (PMC12627406; doi:10.1093/hropen/hoaf069)
Supplement: hoaf069_Supplementary_Data [file hoaf069_supplementary_data.docx]

**SUPPLEMENTARY DATA**

**Supplementary File S1: Detailed experimental processes of whole genome amplification**

**Single-cell whole genome amplification**

The obtained cells underwent whole genome amplification (WGA) and NGS library preparation using the ChromInst^TM^ Universal Library Preparation Kit (Yikon Genomics, Suzhou, China) following the provided operational guidelines. Briefly, after cellular lysis, the liberated genomic DNA was primed with a random primer library, initiating pre-amplification which was succeeded by exponential amplification. The detailed experimental procedures are as follows.

1. **Cell lysis**

1.1 Sample preparation: Thaw the embryo cell/polar body samples at room temperature or use fresh samples. Centrifuge for 30–60 s and then rest the tubes on a tube rack at the correct order before use.

1.2 Positive and Negative controls preparation: Add 1 μl control gDNA (diluted to 50 pg/μl) to a 200 μl centrifuge tube with 5 μl [Sample Preservation Buffer](#samplebuffer) or [Buffer 1](#Buffer1). Label them as a positive or negative control. Use another tube with 6 μl [Sample Preservation Buffer](#samplebuffer) or [Buffer 1](#Buffer1) and label it as a blank control.

1.3 Add 0.5 μl [Cell Lysis Enzyme](#celllysis) onto the inner wall of samples including controls, 3 mm or slightly above the liquid surface (do not insert the pipette tip into the liquid to prevent taking cells away). After that, centrifuge the tubes (30–60 s as recommended). Avoid air bubbles during operation and do not vortex or invert the tubes.

1.4 Perform thermal cycling of the samples with the heated lid turned ON, using the program outlined in **Supplementary Table S2**.

1.5 Stop the program when the lysis process is done. Proceed to the next step immediately.

**2. Pre-amplification**

2.1 Prepare a mix for pre-amplification reaction (**Supplementary Table S3**) considering the number of samples (N). Vortex the mix thoroughly and centrifuge briefly (microcentrifuge, 800 g, 3–5 s).

2.2 Add 30 μl pre-amplification reaction mix into each cell lysis product from step 3.3.5 (the total reaction volume in each tube is about 35 μl at this step). Vortex thoroughly and centrifuge briefly (microcentrifuge, 800 g, 3–5 s).

2.3 Perform thermal cycling of the samples with the heated lid turned ON, using the program outlined in **Supplementary Table S4.**

2.4 Stop the program when the process is done. Proceed to the next step immediately.

**3. Exponential amplification**

3.1 Prepare a mix for exponential amplification reaction (**Supplementary Table S5**) considering the number of samples (N). Vortex the mix thoroughly and centrifuge briefly (microcentrifuge, 800 g, 3–5 s).

3.2 Add 30 μl amplification mix and 1 μl [barcode primer](#barcode) containing the NGS universal index to each pre-amplification reaction product (the total reaction volume in each tube is about 65 μl at this step). Vortex thoroughly and centrifuge briefly (microcentrifuge, 800 g, 3–5 s).

3.3 Perform thermal cycling of the samples with the heated lid turned ON, using the program outlined in **Supplementary Table S6**.

3.4 Stop the program when the process is done.

**Library purification (Optional)**

3.5 Centrifuge the product of Exponential amplification from step 3.5.4 briefly. Then transfer 50 μl of each into a new micro-centrifuge tube and add 50 μl [re-suspended CMPure Magbeads](#beads) (1×, the volume of the beads depends on the volume of the sample) into the same tube. Vortex and mix well, and then centrifuge briefly (microcentrifuge, 800 g, 3–5 s). Incubate at room temperature for 5 min.

3.6 After incubation, place the centrifuge tube from step 3.6.1 on a magnetic stand for about 5 min. When the supernatant becomes clear, while keeping the tube on the magnetic stand, carefully aspirate the supernatant and discard. Do not touch the magnetic beads.

3.7 Add 200 μl freshly prepared 80% ethanol to the tube. Incubate at room temperature for 30 sec, then carefully aspirate the supernatant and discard. DO NOT take the tube off the magnetic stand. DO NOT touch or disrupt the beads during this procedure.

3.8 Repeat 2.3.16 once.

3.9 Keep the tube open on the magnetic stand at room temperature for 5–10 minutes to let the ethanol evaporate and make sure that the beads are dried but not over-dried.

3.10 Add 18 µl [Nuclease-Free Water](#nucleasewater) and close the lids, then resuspend the beads by vortexing (15-30 sec). Pulse centrifuge the tubes and incubate at room temperature for 5 min off the stand.

3.11 Place the tube onto the magnetic stand and wait for about 5 min till the supernatant becomes clear. Carefully transfer 15 μl supernatant to a new 1.5 ml centrifuge tube. DO NOT touch the beads during this procedure.

Validation of the NGS library's quality is carried out through Qubit 3.0 and 1.5% agarose gel electrophoresis. Sequencing is conducted on the Illumina platform, yielding approximately 1.5 million sequencing reads per library.

**Supplementary figures**


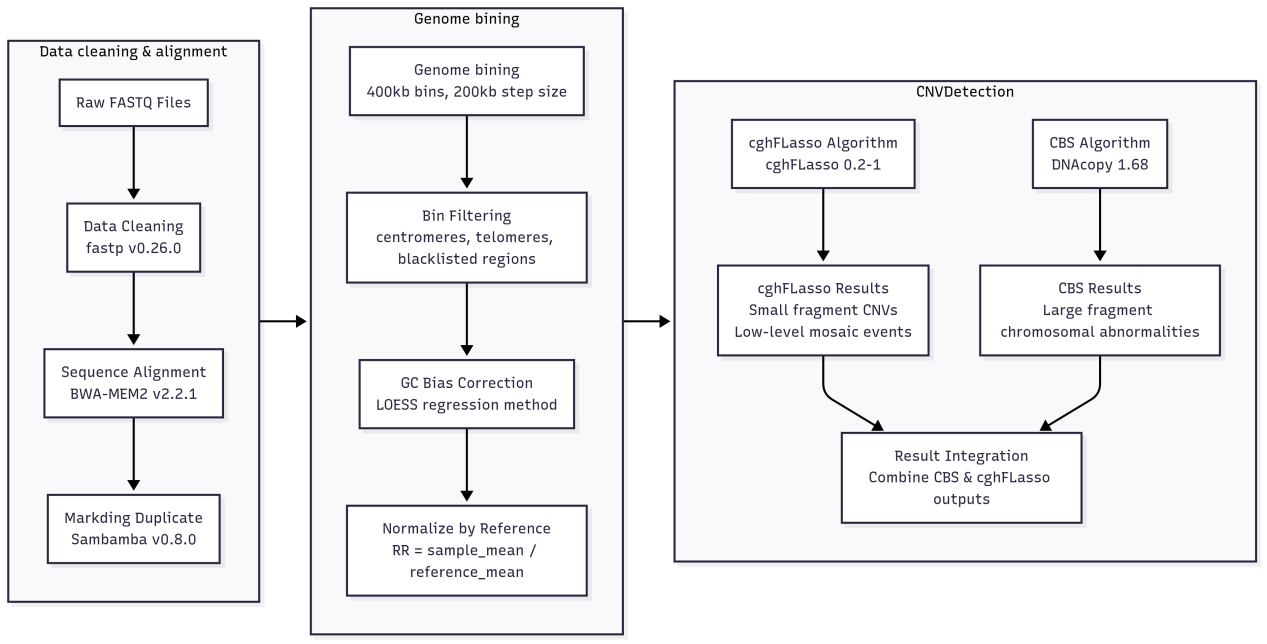


**Supplementary Figure S1. Workflow of copy number variant (CNV) detection based on next-generation sequencing (NGS) and third-generation sequencing (TGS).**

**
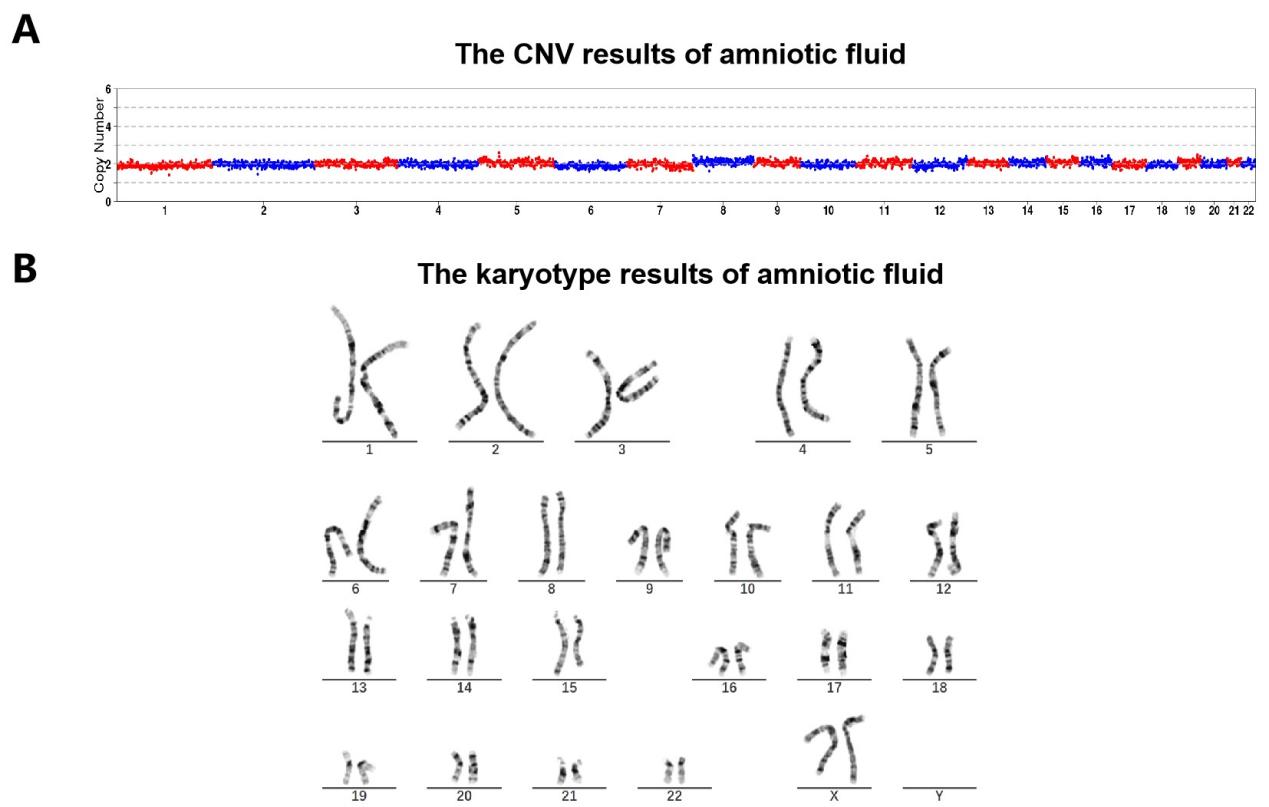
**

**Supplementary Figure S2. Prenatal diagnosis results for PT10. (A)** The copy number variant (CNV) results of amniotic fluid showed 46, XN. **(B)** The karyotype results of amniotic fluid showed 46, XX.

**Supplementary Table S1. Clinical information of all patients.**

| **Female** | | | | **Male** | | |
| --- | --- | --- | --- | --- | --- | --- |
| **Num** | **Age** | **Karyotype** | **Clinical Information** | **Age** | **Karyotype** | **Clinical Information** |
| PT01 | 34 | Normal | \ | 35 | Normal | \ |
| PT02 | 34 | Normal | \ | 47 | Normal | \ |
| PT03 | 35 | Normal | \ | 35 | Inversion on chromosome 2 | \ |
| PT04 | 39 | Normal | \ | 39 | Normal | \ |
| PT05 | 40 | 1.35MB deletion on chromosome 1 | \ | 39 | Normal | \ |
| PT06 | 41 | Normal | RIF | 38 | Normal | \ |
| PT07 | 35 | Normal | \ | 56 | Normal | \ |
| PT08 | 28 | Normal | Carrier of AD genetic diseases | 33 | Normal | \ |
| PT09 | 37 | Normal | RSA | 39 | Normal | \ |
| PT10 | 31 | Normal | RSA | 30 | Normal | \ |
| PT11 | 43 | Normal | AMA | 43 | Normal | Teratozoospermia, sperm supply |
| PT12 | 37 | Normal | RSA | 39 | Normal | \ |
| PT13 | 35 | Normal | RSA | 38 | Normal | \ |
| PT14 | 38 | Normal | RSA | 38 | Normal | \ |
| PT15 | 33 | Normal | heterozygous mutation in the GJB2 | 37 | Normal | heterozygous mutation in the GJB2 |
| PT16 | 29 | 46,XX,t(5;18)(p12;q21) | Balanced translocation | 32 | Normal | \ |
| PT17 | 36 | Normal | RSA | 36 | Normal | \ |
| PT18 | 39 | Normal | RSA, AMA | 41 | Normal | \ |
| PT19 | 38 | Normal | AMA, History of adverse pregnancy | 45 | Normal | \ |
| PT20 | 43 | Normal | AMA, History of adverse pregnancy | 51 | Normal | \ |
| PT21 | 29 | Normal | History of adverse pregnancy | 31 | Normal | 0.18Mb deletion at q33.3 on chromosome 9 |
| PT22 | \ | \ | \ | \ | \ | \ |
| PT23 | 43 | Normal | AMA | 46 | Normal | \ |
| PT24 | 39 | Normal | AMA | 38 | Normal | \ |
| PT25 | 41 | Normal | AMA | 38 | Normal | \ |
| PT26 | 44 | Normal | AMA | 54 | Normal | \ |
| PT27 | \ | \ | \ | \ | \ | \ |
| PT28 | 37 | Normal | RIF | 37 | Normal | \ |
| PT29 | 39 | Normal | AMA | 37 | Normal | \ |
| PT30 | 44 | Normal | AMA | 40 | Normal | \ |

Clinical information includes age, karyotype, and reproductive related clinical phenotypes

**Supplementary Table S2. Thermal cycler program for cell lysis**

| Cycle | Temperature | Time |
| --- | --- | --- |
| 1 | 75°C | 10 min |
|  | 95°C | 4 min |
|  | 22°C | Hold |

**Supplementary Table S3. Preparation of pre-amplification mix**

| Pre-amplification mix | Volume (N=Number of samples) |
| --- | --- |
| [Buffer 2](#Buffer2) | 30 μl × (N+1) |
| [Pre-Lib Enzyme](#prelib) | 1 µl × (N+1) |
| Total volume | 31 µl × (N+1) |

**Supplementary Table S4. Thermal cycler program for pre-amplification**

| Cycle | Temperature | Time |
| --- | --- | --- |
| 1 | 95°C | 2 min |
| 12 | 95°C | 15 s |
|  | 15℃ | 50 s |
|  | 25°C | 40 s |
|  | 35°C | 30 s |
|  | 65°C | 40 s |
|  | 75°C | 40 s |
| 1 | 4°C | Hold |

**Supplementary Table S5. Preparation of exponential amplification mix**

| Pre-amplification mix | Volume (N=Number of samples) |
| --- | --- |
| [Buffer 3](#Buffer3) | 30 μl × (N+1) |
| [Library Enzyme](#lib) | 0.8 µl × (N+1) |
| Total volume | 30.8 µl × (N+1) |

**Supplementary Table S6. Thermal cycler program for exponential amplification**

| Cycle | Temperature | Time |
| --- | --- | --- |
| 1 | 94°C | 30 s |
| 17 | 94°C | 20 s |
|  | 63°C | 30 s |
|  | 72°C | 40 s |
| 1 | 4°C | Hold |

**Supplementary Table S7. Statistical table of amplification concentrations of PBs**

| **Number** | **Sample ID** | **Type of the samples** | **Concentration of the WGA products (ng/μl)** |
| --- | --- | --- | --- |
| 1 | PT01_1 | PB1 | 2.52 |
| 2 | PT01_1 | PB2 | 0.352 |
| 3 | PT01_2 | PB1 | 3.08 |
| 4 | PT01_2 | PB2 | 4.26 |
| 5 | PT01_4 | PB1 | 4.88 |
| 6 | PT01_4 | PB2 | 2.4 |
| 7 | PT01_5 | PB2 | 3.3 |
| 8 | PT01_5 | PB1 | 2.72 |
| 9 | PT01_7 | PB1 | 2.8 |
| 10 | PT01_7 | PB2 | 4.6 |
| 11 | PT01_8 | PB1 | 25.6 |
| 12 | PT01_8 | PB2 | 2.92 |
| 13 | PT01_9 | PB1 | 16.8 |
| 14 | PT01_9 | PB2 | 2.54 |
| 15 | PT01_10 | PB1 | 3.82 |
| 16 | PT01_10 | PB2 | 0.354 |
| 17 | PT01_11 | PB1 | 3.14 |
| 18 | PT01_11 | PB2 | 2.04 |
| 19 | PT02_1 | PB1 | 7.6 |
| 20 | PT02_1 | PB2 | 1.72 |
| 21 | PT02_2 | PB1 | 7.94 |
| 22 | PT02_2 | PB2 | 4.34 |
| 23 | PT03_1 | PB1 | 2.42 |
| 24 | PT03_1 | PB2 | 0.674 |
| 25 | PT03_2 | PB1 | 0.116 |
| 26 | PT03_2 | PB2 | \ |
| 27 | PT03_3 | PB1 | 0.156 |
| 28 | PT03_3 | PB2 | 0.102 |
| 29 | PT03_4 | PB1 | 0.188 |
| 30 | PT03_4 | PB2 | 0.176 |
| 31 | PT04_1 | PB1 | 3.6 |
| 32 | PT04_1 | PB2 | 3.4 |
| 33 | PT04_2 | PB2 | 2.44 |
| 34 | PT04_2 | PB1 | 4.16 |
| 35 | PT04_3 | PB1 | 3.46 |
| 36 | PT04_3 | PB2 | 1.38 |
| 37 | PT05_1 | PB2 | 0.29 |
| 38 | PT05_1 | PB1 | 2.06 |
| 39 | PT05_2 | PB1 | 3.86 |
| 40 | PT05_2 | PB2 | 2.16 |
| 41 | PT05_3 | PB2 | 1.51 |
| 42 | PT05_3 | PB1 | 2.74 |
| 43 | PT05_4 | PB2 | 1.46 |
| 44 | PT05_4 | PB1 | 4.46 |
| 45 | PT05_5 | PB2 | 0.434 |
| 46 | PT05_5 | PB1 | 3.6 |
| 47 | PT05_6 | PB1 | 4.16 |
| 48 | PT05_6 | PB2 | 1.46 |
| 49 | PT05_7 | PB1 | 3.52 |
| 50 | PT05_7 | PB2 | 1.94 |
| 51 | PT05_8 | PB1 | 3.78 |
| 52 | PT05_8 | PB2 | 0.288 |
| 53 | PT05_9 | PB1 | 1.76 |
| 54 | PT05_9 | PB2 | 1.25 |
| 55 | PT06_2 | PB2 | 1.4 |
| 56 | PT06_2 | PB1 | 0.452 |
| 57 | PT06_6 | PB1+PB2 | 5.8 |
| 58 | PT06_8 | PB1 | 4.76 |
| 59 | PT06_8 | PB2 | 3.36 |
| 60 | PT07_1 | PB1 | 3.62 |
| 61 | PT07_1 | PB2 | 3 |
| 62 | PT07_3 | PB1 | 6.3 |
| 63 | PT07_3 | PB2 | 3.02 |
| 64 | PT07_5 | PB1 (Contamination) | 6.72 |
| 65 | PT07_5 | PB2 | 0.41 |
| 66 | PT07_6 | PB1 | 4.06 |
| 67 | PT07_6 | PB2 | 2.34 |
| 68 | PT07_7 | PB1 | 2.54 |
| 69 | PT07_7 | PB2 | 4.78 |
| 70 | PT08_4 | PB1 | 4.38 |
| 71 | PT08_4 | PB2 | 2.9 |
| 72 | PT08_5 | PB1 | 6.74 |
| 73 | PT08_5 | PB2 | 2.36 |
| 74 | PT08_6 | PB1 | 4.1 |
| 75 | PT08_6 | PB2 | 2.84 |
| 76 | PT08_8 | PB2 | 2.38 |
| 77 | PT08_8 | PB1 | 5.16 |
| 78 | PT09_1 | PB1+PB2 | 10.6 |
| 79 | PT09_2 | PB2 (Contamination) | 1.53 |
| 80 | PT09_2 | PB1 | 1.35 |
| 81 | PT09_3 | PB1 | 1.88 |
| 82 | PT09_3 | PB2 (Contamination) | 1.86 |
| 83 | PT09_4 | PB1+PB2 | 9.38 |
| 84 | PT09_6 | PB1+PB2 | 1.53 |
| 85 | PT09_7 | PB1+PB2 | 12 |
| 86 | PT10_1 | PB1 | 3.2 |
| 87 | PT10_1 | PB2 | 2.82 |
| 88 | PT10_2 | PB1 | 3.56 |
| 89 | PT10_2 | PB2 (Contamination) | 1.91 |
| 90 | PT10_3 | PB1 | 3.56 |
| 91 | PT10_3 | PB2 | 2.5 |
| 92 | PT10_4 | PB1 | 5.48 |
| 93 | PT10_4 | PB2 | 4.12 |
| 94 | PT11_1 | PB1 | 5.36 |
| 95 | PT11_1 | PB2 | 1.82 |
| 96 | PT11_2 | PB1 | 4.12 |
| 97 | PT11_2 | PB2 | 2.72 |
| 98 | PT12_2 | PB1 | 5.32 |
| 99 | PT12_2 | PB2 | 3.64 |
| 100 | PT12_3 | PB1 | 3.3 |
| 101 | PT12_3 | PB2 (Contamination) | 4.38 |
| 102 | PT12_5 | PB1 | 6.28 |
| 103 | PT12_5 | PB2 | 4.58 |
| 104 | PT12_6 | PB1 | 5.18 |
| 105 | PT12_6 | PB2 | 4.58 |
| 106 | PT13_1 | PB1 | 6.04 |
| 107 | PT13_1 | PB2 | 5.58 |
| 108 | PT13_2 | PB1 | 8.5 |
| 109 | PT13_2 | PB2 | 1.98 |
| 110 | PT13_4 | PB1 | 4.18 |
| 111 | PT13_4 | PB2 | 2.78 |
| 112 | PT13_5 | PB1 | 2.24 |
| 113 | PT13_5 | PB2 | 3.26 |
| 114 | PT13_6 | PB1 | 0.72 |
| 115 | PT13_6 | PB2 | 0.644 |
| 116 | PT14_1 | PB2 | 0.998 |
| 117 | PT14_1 | PB1 | 2.08 |
| 118 | PT14_2 | PB2 | 1.77 |
| 119 | PT14_2 | PB1 | 2.46 |
| 120 | PT14_3 | PB2 | 4 |
| 121 | PT14_3 | PB1 | 3.18 |
| 122 | PT14_4 | PB2 | 2.6 |
| 123 | PT14_4 | PB1 | 3.56 |
| 124 | PT14_5 | PB2 | 1.71 |
| 125 | PT14_5 | PB1 | 4.24 |
| 126 | PT14_6 | PB2 | 2.42 |
| 127 | PT14_6 | PB1 | 3.06 |
| 128 | PT15_1 | PB1 | 0.538 |
| 129 | PT15_1 | PB2 | 0.486 |
| 130 | PT15_2 | PB1 | 1.17 |
| 131 | PT15_2 | PB2 | 0.338 |
| 132 | PT15_4 | PB1 | 1.25 |
| 133 | PT15_4 | PB2 | 0.42 |
| 134 | PT15_5 | PB1 | 0.762 |
| 135 | PT15_5 | PB2 | 0.866 |
| 136 | PT15_6 | PB1 | 0.912 |
| 137 | PT15_6 | PB2 | 0.51 |
| 138 | PT16_1 | PB1 | 5.82 |
| 139 | PT16_1 | PB2 | 6.7 |
| 140 | PT16_2 | PB1 | 6.96 |
| 141 | PT16_2 | PB2 | 5.16 |
| 142 | PT16_3 | PB1 | 4.6 |
| 143 | PT16_3 | PB2 | 6.08 |
| 144 | PT16_4 | PB1 | 3.58 |
| 145 | PT16_4 | PB2 | 8.12 |
| 146 | PT16_5 | PB1 | 9.82 |
| 147 | PT16_5 | PB2 | 4.92 |
| 148 | PT16_6 | PB1 | 3.74 |
| 149 | PT16_6 | PB2 | 4.92 |
| 150 | PT16_7 | PB1 | 4.84 |
| 151 | PT16_7 | PB2 (Contamination) | 2.02 |
| 152 | PT16_8 | PB1 | 10.2 |
| 153 | PT16_8 | PB2 | 4.86 |
| 154 | PT16_10 | PB1 | 4.66 |
| 155 | PT16_10 | PB2 | 7.56 |
| 156 | PT16_11 | PB1 | 2.66 |
| 157 | PT16_11 | PB2 | 7.4 |
| 158 | PT16_13 | PB1 | 6.86 |
| 159 | PT16_13 | PB2 | 3.2 |
| 160 | PT16_14 | PB1 | 1.36 |
| 161 | PT16_14 | PB2 | 3.36 |
| 162 | PT16_15 | PB1 | 7.4 |
| 163 | PT16_15 | PB2 | 2.44 |
| 164 | PT16_16 | PB1 (Contamination) | 8.46 |
| 165 | PT16_16 | PB2 | 3.16 |
| 166 | PT17_1 | PB1+PB2 | 6.38 |
| 167 | PT17_3 | PB1+PB2 | 5.58 |
| 168 | PT17_5 | PB1+PB2 | 2.08 |
| 169 | PT17_8 | PB1+PB2 | 2.8 |
| 170 | PT17_11 | PB1+PB2 | 3.2 |
| 171 | PT17_12 | PB1+PB2 | 3.62 |
| 172 | PT17_13 | PB1+PB2 | 5.86 |
| 173 | PT17_14 | PB1+PB2 | 13.1 |
| 174 | PT18_2 | PB1+PB2 | 6.24 |
| 175 | PT19_1 | PB1+PB2 | 4.64 |
| 176 | PT22_1 | PB1 | 1.43 |
| 177 | PT22_1 | PB2 | 2.22 |
| 178 | PT22_2 | PB1 | 2.68 |
| 179 | PT22_2 | PB2 | 2.22 |
| 180 | PT22_3 | PB1 | 2.9 |
| 181 | PT22_3 | PB2 | 3.32 |
| 182 | PT22_4 | PB1 | 3.48 |
| 183 | PT22_4 | PB2 | 3.96 |
| 184 | PT22_5 | PB1 | 2.76 |
| 185 | PT22_5 | PB2 | 1.66 |
| 186 | PT22_7 | PB1 | 7.14 |
| 187 | PT22_7 | PB2 | 2.8 |
| 188 | PT22_8 | PB1+PB2 | 5.38 |
| 189 | PT22_9 | PB1 | 2.78 |
| 190 | PT22_9 | PB2 | 1.95 |
| 191 | PT23_2 | PB1+PB2 | 9.64 |
| 192 | PT23_3 | PB1+PB2 | 8.8 |
| 193 | PT23_4 | PB1+PB2 | 6.8 |
| 194 | PT23_5 | PB1+PB2 | 10.7 |
| 195 | PT23_6 | PB1+PB2 | 7.36 |
| 196 | PT24_1 | PB1+PB2 | 7.86 |
| 197 | PT24_2 | PB1+PB2 | 12.8 |
| 198 | PT25_2 | PB1+PB2 | 9.28 |
| 199 | PT25_4 | PB1+PB2 | 10.9 |
| 200 | PT26_4 | PB1+PB2 | 7.6 |
| 201 | PT27_2 | PB1 | 4.34 |
| 202 | PT27_2 | PB2 | 1.4 |
| 203 | PT27_4 | PB1 | 3.72 |
| 204 | PT27_4 | PB2 | 2.1 |
| 205 | PT28_1 | PB1+PB2 | 7.26 |
| 206 | PT28_2 | PB1+PB2 | 6.8 |
| 207 | PT28_3 | PB1+PB2 | 5.6 |
| 208 | PT29_1 | PB1+PB2 | 4.94 |
| 209 | PT29_2 | PB1+PB2 | 3.9 |
| 210 | PT29_3 | PB1+PB2 | 6.52 |
| 211 | PT29_4 | PB1+PB2 | 3.86 |
| 212 | PT29_5 | PB1+PB2 | 5.54 |
| 213 | PT29_6 | PB1+PB2 | 2.22 |
| 214 | PT30_12 | PB1+PB2 | 6.62 |

**Supplementary Table S8. All copy number variant (CNV) results for PBs, including next-generation sequencing (NGS) and third-generation sequencing (TGS)-based results**

| **Num** | **Sample ID** | **Type of the samples** | **CNV based on TGS** | **CNV based on NGS** |
| --- | --- | --- | --- | --- |
|  |  |  |  |  |
| 1 | PT01_1 | PB1 | Euploidy | Euploidy |
| 2 | PT01_1 | PB2 | N/A | N/A |
| 3 | PT01_2 | PB1 | dup(1)(q21.1q44)(~104.75Mb) | dup(1)(q21.1q44)(~102.85Mb) |
| 4 | PT01_2 | PB2 | del(1)(q21.1q44)(~102.85Mb),+16,+22 | del(1)(q21.1q44)(~102.85Mb),+16,+22 |
| 5 | PT01_4 | PB1 | Euploidy | Euploidy |
| 6 | PT01_4 | PB2 | +20,-21 | +20,-21 |
| 7 | PT01_5 | PB1 | Euploidy | Euploidy |
| 8 | PT01_5 | PB2 | -1(~44%),-3(~43%),-6(~44%),-8(~44%) | -1(~43%),-3(~43%),-6(~44%),-8(~44%) |
| 9 | PT01_7 | PB1 | Euploidy | Euploidy |
| 10 | PT01_7 | PB2 | Euploidy | Euploidy |
| 11 | PT01_8 | PB1 | Euploidy | Euploidy |
| 12 | PT01_8 | PB2 | Euploidy | Euploidy |
| 13 | PT01_9 | PB1 | Euploidy | Euploidy |
| 14 | PT01_9 | PB2 | Euploidy | Euploidy |
| 15 | PT01_10 | PB1 | Euploidy | Euploidy |
| 16 | PT01_10 | PB2 | N/A | N/A |
| 17 | PT01_11 | PB1 | Euploidy | Euploidy |
| 18 | PT01_11 | PB2 | Euploidy | Euploidy |
| 19 | PT02_1 | PB1 | +19 | +19 |
| 20 | PT02_1 | PB2 | N/A | N/A |
| 21 | PT02_2 | PB1 | -3,-5,-7,-8,-13,-15,-16,-19,-21,-22 | -3,-5,-7,-8,-13,-15,-16,-19,-21,-22 |
| 22 | PT02_2 | PB2 | Euploidy | Euploidy |
| 23 | PT03_1 | PB1 | \ | +7,-9,+10,-11,+14 |
| 24 | PT03_1 | PB2 | \ | N/A |
| 25 | PT03_2 | PB1 | \ | -15 |
| 26 | PT03_2 | PB2 | \ | +15,+19 |
| 27 | PT03_3 | PB1 | \ | Euploidy |
| 28 | PT03_3 | PB2 | \ | Euploidy |
| 29 | PT03_4 | PB1 | \ | Euploidy |
| 30 | PT03_4 | PB2 | \ | Euploidy |
| 31 | PT04_1 | PB1 | Euploidy | Euploidy |
| 32 | PT04_1 | PB2 | Euploidy | Euploidy |
| 33 | PT04_2 | PB1 | +5,+14,-15 | +5,+14,-15 |
| 34 | PT04_2 | PB2 | -5,-14,+16,-22(~33%) | -5,-14,+16 |
| 35 | PT04_3 | PB1 | +X | +X |
| 36 | PT04_3 | PB2 | Euploidy | Euploidy |
| 37 | PT05_1 | PB1 | Euploidy | Euploidy |
| 38 | PT05_1 | PB2 | -21 | -21 |
| 39 | PT05_2 | PB1 | +13 | +13 |
| 40 | PT05_2 | PB2 | dup(7)(q31.1)(~4.00Mb), dup(8)(q22.1q22.2)(~4.60Mb) | dup(7)(q31.1)(~4.20Mb) |
| 41 | PT05_3 | PB1 | Euploidy | Euploidy |
| 42 | PT05_3 | PB2 | Euploidy | Euploidy |
| 43 | PT05_4 | PB1 | Euploidy | Euploidy |
| 44 | PT05_4 | PB2 | Euploidy | Euploidy |
| 45 | PT05_5 | PB1 | +12 | +12 |
| 46 | PT05_5 | PB2 | N/A | N/A |
| 47 | PT05_6 | PB1 | +16 | +16 |
| 48 | PT05_6 | PB2 | +22 | +22 |
| 49 | PT05_7 | PB1 | Euploidy | Euploidy |
| 50 | PT05_7 | PB2 | dup(19)(q13.2q13.43)(~19.73Mb,~55%) | Euploidy |
| 51 | PT05_8 | PB1 | +3(~64%),-15(~66%) | +3(~60%),-15(~63%) |
| 52 | PT05_8 | PB2 | N/A | N/A |
| 53 | PT05_9 | PB1 | Euploidy | Euploidy |
| 54 | PT05_9 | PB2 | dup(16)(p11.2)(~4.00Mb), dup(19)(p13.3p13.11)(~14.60Mb,~66%) | dup(1)(p36.12p35.1)(~11.40Mb,~47%) |
| 55 | PT06_2 | PB1 | N/A | N/A |
| 56 | PT06_2 | PB2 | +17,-22 | +17,-22 |
| 57 | PT06_6 | PB1+PB2 | Euploidy | Euploidy |
| 58 | PT06_8 | PB1 | -21 | -21 |
| 59 | PT06_8 | PB2 | +16,-22 | +16,-22 |
| 60 | PT07_1 | PB1 | -2,+3,+4,+5,+6,-7,-9,+10,-11, +12,-14,-15,-16,-18,+19,+20,-21 | -2,+3,+4,+5,+6,-7,-9,+10,-11, +12,-14,-15,-16,-18,+19,+20,-21 |
| 61 | PT07_1 | PB2 | del(6)(p25.3p21.2)(~38.00Mb,~39%), del(21)(q21.3q22.3)(~20.93Mb,~39%) | del(21)(q21.3q22.3)(~16.93Mb,~48%) |
| 62 | PT07_3 | PB1 | dup(4)(p16.3p16.1)(~7.20Mb), dup(19)(p13.3p13.2)(~11.60Mb), dup(19)(q11q13.31)(~16.00Mb), dup(19)(q13.33q13.41)(~4.00Mb), dup(19)(q13.41q13.43)(~6.13Mb) | dup(4)(p16.3p16.1)(~9.20Mb), dup(4)(q28.2q28.3)(~4.00Mb), +19 |
| 63 | PT07_3 | PB2 (Contamination) | del(5)(q15q35.3)(~86.92Mb,~56%) | del(5)(q15q35.3)(~86.92Mb,~56%) |
| 64 | PT07_5 | PB1 (Contamination) | -X(~45%)(10% Y contamination) | -X(~53%)(10% Y contamination) |
| 65 | PT07_5 | PB2 | N/A | N/A |
| 66 | PT07_6 | PB1 | +11,del(17)(p13.3p11.2)(~19.60Mb) | +11,del(17)(p13.3p11.2)(~19.20Mb) |
| 67 | PT07_6 | PB2 | dup(17)(p13.3p11.2)(~19.20Mb) | dup(17)(p13.3p11.2)(~19.00Mb) |
| 68 | PT07_7 | PB1 | -5(~48%) | -5(~47%) |
| 69 | PT07_7 | PB2 (Contamination) | Euploidy | Euploidy (10% Y contamination) |
| 70 | PT08_4 | PB1 | Euploidy | Euploidy |
| 71 | PT08_4 | PB2 | Euploidy | Euploidy |
| 72 | PT08_5 | PB1 | Euploidy | Euploidy |
| 73 | PT08_5 | PB2 | Euploidy | Euploidy |
| 74 | PT08_6 | PB1 | Euploidy | Euploidy |
| 75 | PT08_6 | PB2 | Euploidy | Euploidy |
| 76 | PT08_8 | PB1 | Euploidy | Euploidy |
| 77 | PT08_8 | PB2 | Euploidy | Euploidy |
| 78 | PT09_1 | PB1+PB2 | Euploidy | Euploidy |
| 79 | PT09_2 | PB2  (Contamination) | dup(5)(q35.2q35.3)(~4.72Mb),dup(19)(q13.41q13.43)(~5.73Mb)(10% Y contamination) | Euploidy (10% Y contamination) |
| 80 | PT09_2 | PB1 | Euploidy | Euploidy |
| 81 | PT09_3 | PB1 | N/A | Euploidy |
| 82 | PT09_3 | PB2  (Contamination) | Euploidy (10% Y contamination) | Euploidy (10% Y contamination) |
| 83 | PT09_4 | PB1+PB2 | Euploidy | Euploidy |
| 84 | PT09_6 | PB1+PB2 | Euploidy | Euploidy |
| 85 | PT09_7 | PB1+PB2 | Euploidy | Euploidy |
| 86 | PT10_1 | PB1 | Euploidy | \ |
| 87 | PT10_1 | PB2 | Euploidy | \ |
| 88 | PT10_2 | PB1 | Euploidy | \ |
| 89 | PT10_2 | PB2  (Contamination) | Euploidy(10% Y contamination) | \ |
| 90 | PT10_3 | PB1 | Euploidy | \ |
| 91 | PT10_3 | PB2 | Euploidy | \ |
| 92 | PT10_4 | PB1 | 16 | \ |
| 93 | PT10_4 | PB2 | dup(6)(p22.2p21.2)(~12.60Mb,~41%), -16, dup(17)(q11.1q21.31)(~18.80Mb,~31%) ,+19(~36%) | \ |
| 94 | PT11_1 | PB1 | +3,+5,+6,+15,-21 | \ |
| 95 | PT11_1 | PB2 | dup(1)(q21.1q44)(~104.45Mb),+2,-3,-4,-6,+7,+8,+9,-12,+13,-15,+16,-17,+19,-20,+21 | \ |
| 96 | PT11_2 | PB1 | -X | \ |
| 97 | PT11_2 | PB2 | dup(19)(q13.31q13.33)(~5.40Mb) | \ |
| 98 | PT12_2 | PB1 | +13 | +13 |
| 99 | PT12_2 | PB2 | Euploidy | Euploidy |
| 100 | PT12_3 | PB1 | +21 | del(1)(q21.1q44)(~104.45Mb,~48%),+21 |
| 101 | PT12_3 | PB2  (Contamination) | Euploidy(10% Y contamination) | Euploidy(10% Y contamination) |
| 102 | PT12_5 | PB1 | Euploidy | Euploidy |
| 103 | PT12_5 | PB2 | -21 | -21 |
| 104 | PT12_6 | PB1 | Euploidy | Euploidy |
| 105 | PT12_6 | PB2 | Euploidy | Euploidy |
| 106 | PT13_1 | PB1 | Euploidy | Euploidy |
| 107 | PT13_1 | PB2 | -15 | -15 |
| 108 | PT13_2 | PB1 | Euploidy | Euploidy |
| 109 | PT13_2 | PB2 | Euploidy | Euploidy |
| 110 | PT13_4 | PB1 | Euploidy | Euploidy |
| 111 | PT13_4 | PB2 | Euploidy | Euploidy |
| 112 | PT13_5 | PB1 | del(9)(p24.3p23)(~9.60Mb), del(9)(p21.1p13.1)(~8.00Mb) -13,-14(~48%),-16,+21(~57%) | del(9)(p24.3p13.1)(~39.00Mb,~51%), del(13)(q11q21.1)(~36.00Mb), del(13)(q14.3q34)(~59.97Mb,~50%), -14(~49%),-16,+21(~54%) |
| 113 | PT13_5 | PB2 | -21 | -21 |
| 114 | PT13_6 | PB1 | Euploidy | Euploidy |
| 115 | PT13_6 | PB2 | Euploidy | Euploidy |
| 116 | PT14_1 | PB1 | Euploidy | Euploidy |
| 117 | PT14_1 | PB2 | -17 | -17 |
| 118 | PT14_2 | PB1 | Euploidy | Euploidy |
| 119 | PT14_2 | PB2 | Euploidy | Euploidy |
| 120 | PT14_3 | PB1 | Euploidy | Euploidy |
| 121 | PT14_3 | PB2 | Euploidy | Euploidy |
| 122 | PT14_4 | PB1 | Euploidy | Euploidy |
| 123 | PT14_4 | PB2 | -16 | -16 |
| 124 | PT14_5 | PB1 | Euploidy | Euploidy |
| 125 | PT14_5 | PB2 | Euploidy | Euploidy |
| 126 | PT14_6 | PB1 | Euploidy | Euploidy |
| 127 | PT14_6 | PB2  (Contamination) | dup(6)(q22.31q27)(~50.92Mb), dup(8)(p23.3p11.1)(~43.60Mb), dup(15)(q25.2q26.3)(~19.53Mb,~52%) dup(17)(q24.3q25.3)(~6.20Mb) | dup(6)(q22.31q27)(~50.92Mb), dup(15)(q25.2q26.3)(~19.53Mb,~51%), dup(17)(q24.3q25.3)(~10.40Mb) |
| 128 | PT15_1 | PB1 | \ | Euploidy |
| 129 | PT15_1 | PB2 | \ | Euploidy |
| 130 | PT15_2 | PB1 | \ | Euploidy |
| 131 | PT15_2 | PB2 | \ | Euploidy |
| 132 | PT15_4 | PB1 | \ | dup(17)(p13.3p11.2)(~16.80Mb) |
| 133 | PT15_4 | PB2 | \ | del(17)(p13.3p11.2)(~16.80Mb), dup(17)(p11.2q25.3)(~62.60Mb) |
| 134 | PT15_5 | PB1 | \ | -X,-1,-6,-8,-12 |
| 135 | PT15_5 | PB2 | \ | +X,+1,+6,+8,+12 |
| 136 | PT15_6 | PB1 | \ | -17,-22(~64%) |
| 137 | PT15_6 | PB2 | \ | Euploidy |
| 138 | PT16_1 | PB1 | del(5)(q13.2q35.3)(~110.32Mb),del(18)(p11.32q21.33)(~61.00Mb,~61%),dup(18)(q21.33q23)(~17.28Mb),-20(~39%) | del(5)(q13.2q35.3)(~109.92Mb),del(12)(q24.22q24.31)(~5.20Mb),del(18)(p11.32q21.33)(~60.20Mb,~60%),dup(18)(q21.33q23)(~17.28Mb),-20(~38%) |
| 139 | PT16_1 | PB2 | del(5)(p15.33p15.31)(~6.20Mb),del(5)(p14.3p11)(~27.60Mb),del(5)(q11.1q11.2)(~4.20Mb),del(5)(q12.1q13.2)(~12.20Mb),dup(5)(q14.2q35.3)(~99.52Mb,~38%),del(8)(p11.22p11.1)(~5.20Mb),del(8)(q11.1q21.11)(~27.20Mb,~55%),-16,del(17)(p13.2p11.2)(~13.40Mb,~56%),del(17)(q21.2q21.31)(~4.00Mb),del(17)(q21.33q22)(~7.00Mb),del(17)(q23.1q25.3)(~23.40Mb),del(18)(q21.33q23)(~17.28Mb) | del(5)(p15.33p14.1)(~28.40Mb,~49%),del(5)(p14.1p13.1)(~11.80Mb),del(5)(p13.1p11)(~5.60Mb),del(5)(q11.1q13.2)(~22.40Mb),dup(5)(q14.3q35.3)(~94.20Mb,~33%),del(8)(p12p11.1)(~13.40Mb,~56%),del(8)(q11.1q21.11)(~27.20Mb,~53%),del(11)(p14.3p14.1)(~4.40Mb),-16,del(17)(p13.1p11.2)(~10.80Mb,~61%),del(17)(q12q21.33)(~10.80Mb),del(17)(q21.33q22)(~7.00Mb),del(17)(q23.1q25.3)(~23.40Mb),del(18)(q21.33q23)(~17.08Mb) |
| 140 | PT16_2 | PB1 | dup(5)(q13.2q35.3)(~109.52Mb),del(18)(q21.33q23)(~17.28Mb) | dup(5)(q13.2q35.3)(~109.72Mb),del(18)(q21.33q23)(~17.28Mb) |
| 141 | PT16_2 | PB2 | del(5)(q13.2q35.3)(~109.52Mb),dup(18)(q21.33q23)(~17.08Mb) | del(5)(q13.2q35.3)(~109.52Mb),dup(18)(q21.33q23)(~17.28Mb) |
| 142 | PT16_3 | PB1 | Euploidy | Euploidy |
| 143 | PT16_3 | PB2 | dup(5)(q13.2q35.3)(~109.52Mb),del(18)(q21.33q23)(~17.08Mb) | dup(5)(q13.2q35.3)(~109.52Mb),del(18)(q21.33q23)(~17.08Mb) |
| 144 | PT16_4 | PB1 | +19 | +19 |
| 145 | PT16_4 | PB2 | dup(5)(q13.2q35.3)(~109.52Mb),del(18)(q21.33q23)(~17.08Mb) | dup(5)(q13.2q35.3)(~109.52Mb),del(18)(q21.33q23)(~17.08Mb) |
| 146 | PT16_5 | PB1 | Euploidy | Euploidy |
| 147 | PT16_5 | PB2 | del(5)(q13.2q35.3)(~109.52Mb),dup(18)(q21.33q23)(~17.08Mb) | del(5)(q13.2q35.3)(~109.52Mb),dup(18)(q21.33q23)(~17.08Mb) |
| 148 | PT16_6 | PB1 | +1 | +1 |
| 149 | PT16_6 | PB2 | -1,dup(6)(q23.2q24.1)(~7.40Mb,~70%),dup(6)(q25.1q27)(~21.12Mb,~50%) | -1,dup(6)(q23.2q27)(~37.72Mb,~42%) |
| 150 | PT16_7 | PB1 | dup(5)(q13.2q35.3)(~109.52Mb),+16,del(18)(q21.33q23)(~17.28Mb) | dup(5)(q13.2q35.3)(~109.52Mb),+16,del(18)(q21.33q23)(~17.28Mb) |
| 151 | PT16_7 | PB2  (Contamination) | Euploidy | Euploidy |
| 152 | PT16_8 | PB1 | del(5)(p15.33q13.2)(~71.80Mb),del(16)(q21q24.3)(~24.75Mb),dup(18)(p11.32q21.33)(~61.20Mb) | del(5)(p15.33q13.2)(~71.80Mb),del(16)(q21q24.3)(~24.75Mb), dup(18)(p11.32q21.33)(~61.20Mb) |
| 153 | PT16_8 | PB2 | +5,dup(16)(q21q22.1)(~4.60Mb),dup(16)(q23.2q24.1)(~5.00Mb),-18 | +5,dup(16)(q21q22.1)(~4.20Mb),dup(16)(q23.2q24.3)(~8.60Mb), dup(17)(q12q21.33)(~15.20Mb,~37%),-18 |
| 154 | PT16_10 | PB1 | dup(5)(p15.33q13.2)(~71.60Mb),del(18)(p11.32q21.33)(~61.20Mb) | dup(5)(p15.33q13.2)(~71.80Mb),del(18)(p11.32q21.33)(~61.20Mb) |
| 155 | PT16_10 | PB2 | del(5)(p15.33q13.2)(~71.60Mb),dup(18)(p11.32q21.33)(~61.20Mb) | del(5)(p15.33q13.2)(~71.60Mb),dup(18)(p11.32q21.33)(~61.20Mb) |
| 156 | PT16_11 | PB1 | del(5)(q13.2q35.3)(~109.52Mb),dup(18)(q21.33q23)(~16.88Mb) | del(5)(q13.2q35.3)(~109.52Mb),dup(18)(q21.33q23)(~17.08Mb) |
| 157 | PT16_11 | PB2 | Euploidy | Euploidy |
| 158 | PT16_13 | PB1 | dup(5)(p15.33q13.2)(~71.60Mb),del(18)(p11.32q21.33)(~61.20Mb) | dup(5)(p15.33q13.2)(~71.60Mb),del(18)(p11.32q21.33)(~61.20Mb) |
| 159 | PT16_13 | PB2 | del(5)(p15.33q13.2)(~71.40Mb),dup(5)(q13.2q35.3)(~109.32Mb),dup(18)(p11.32q21.33)(~61.20Mb),del(18)(q21.33q23)(~17.08Mb) | del(5)(p15.33q13.2)(~71.40Mb),dup(5)(q13.2q35.3)(~109.32Mb), dup(18)(p11.32q21.33)(~61.20Mb),del(18)(q21.33q23)(~17.08Mb) |
| 160 | PT16_14 | PB1 | del(5)(q13.2q35.3)(~109.52Mb),del(16)(p13.3p13.11)(~16.40Mb),dup(18)(q21.33q23)(~17.08Mb) | del(5)(q13.2q35.3)(~109.52Mb),del(16)(p13.3p13.11)(~16.40Mb) ,dup(18)(q21.33q23)(~17.08Mb) |
| 161 | PT16_14 | PB2 | dup(5)(q13.2q35.3)(~109.72Mb),dup(16)(p13.3p13.11)(~16.40Mb),dup(18)(p11.32q21.33)(~61.20Mb) | dup(5)(q13.2q35.3)(~109.52Mb),dup(16)(p13.3p13.11)(~16.40Mb), dup(18)(p11.32q21.33)(~61.00Mb) |
| 162 | PT16_15 | PB1 | dup(5)(q13.2q35.3)(~109.52Mb),del(18)(q21.33q23)(~17.28Mb) | dup(5)(q13.2q35.3)(~109.52Mb),del(18)(q21.33q23)(~17.28Mb) |
| 163 | PT16_15 | PB2 | del(5)(q13.2q35.3)(~109.52Mb),dup(18)(q21.33q23)(~17.28Mb) | del(5)(q13.2q35.3)(~109.52Mb),dup(18)(q21.33q23)(~17.28Mb) |
| 164 | PT16_16 | PB1  (Contamination) | del(5)(p15.33q13.2)(~71.80Mb),dup(7)(q21.13q31.1)(~18.40Mb),dup(18)(p11.32q21.33)(~60.00Mb),dup(18)(q21.33q23)(~18.28Mb,~37%),+21(~56%)(10% Y contamination) | dup(2)(q32.1q32.2)(~6.00Mb),del(5)(p15.33q13.2)(~71.80Mb) +7(~41%),dup(12)(q15q21.2)(~5.80Mb),dup(18)(p11.32q21.33)(~59.80Mb), dup(18)(q21.33q23)(~18.48Mb,~42%),+21(~44%),+22(~47%)(10% Y contamination) |
| 165 | PT16_16 | PB2 | dup(5)(p15.33q13.2)(~71.60Mb),del(18)(p11.32q21.33)(~61.20Mb) | dup(5)(p15.33q13.2)(~71.80Mb),del(18)(p11.32q21.33)(~61.20Mb) |
| 166 | PT17_1 | PB1+PB2 | Euploidy | Euploidy |
| 167 | PT17_3 | PB1+PB2 | Euploidy | Euploidy |
| 168 | PT17_5 | PB1+PB2 | Euploidy | Euploidy |
| 169 | PT17_8 | PB1+PB2 | Euploidy | Euploidy |
| 170 | PT17_11 | PB1+PB2 | Euploidy | Euploidy |
| 171 | PT17_12 | PB1+PB2 | Euploidy | Euploidy |
| 172 | PT17_13 | PB1+PB2 | Euploidy | Euploidy |
| 173 | PT17_14 | PB1+PB2 | Euploidy | Euploidy |
| 174 | PT18_2 | PB1+PB2 | Euploidy | Euploidy |
| 175 | PT19_1 | PB1+PB2 | -6(~45%),-10(~45%) | del(6)(p25.3p11.2)(~58.00Mb) |
| 176 | PT22_1 | PB1 | N/A | N/A |
| 177 | PT22_1 | PB2 | Euploidy | Euploidy |
| 178 | PT22_2 | PB1 | N/A | -3(~30%),dup(4)(p16.3p16.1)(~10.20Mb),+6,-14(~30%),+20 |
| 179 | PT22_2 | PB2 | Euploidy | Euploidy |
| 180 | PT22_3 | PB1 | +16,-19 | dup(10)(q26.2q26.3)(~5.33Mb),+16,-19 |
| 181 | PT22_3 | PB2 | Euploidy | Euploidy |
| 182 | PT22_4 | PB1 | Euploidy | Euploidy |
| 183 | PT22_4 | PB2 | Euploidy | Euploidy |
| 184 | PT22_5 | PB1 | Euploidy | Euploidy |
| 185 | PT22_5 | PB2 | Euploidy | Euploidy |
| 186 | PT22_7 | PB1 | +8,+13 | +8,+13 |
| 187 | PT22_7 | PB2 | -8,-13 | -8,-13 |
| 188 | PT22_8 | PB1+PB2 | Euploidy | Euploidy |
| 189 | PT22_9 | PB1 | dup(11)(p11.2)(~4.20Mb),dup(11)(q12.1q12.3)(~4.40Mb),-13,dup(19)(q13.42q13.43)(~4.93Mb) | +11(~33%),-13,dup(19)(q13.42q13.43)(~4.93Mb) |
| 190 | PT22_9 | PB2 | dup(4)(p16.3p11)(~49.20Mb,~32%),dup(6)(q25.1q27)(~20.12Mb,~69%),dup(7)(q11.21q11.22)(~6.20Mb),dup(9)(q22.32q31.1)(~8.60Mb),dup(19)(q13.32q13.33)(~4.80Mb),+20(~43%),dup(22)(q11.1q11.21)(~4.40Mb) | dup(4)(p16.1p15.32)(~5.60Mb),dup(6)(q24.3q25.3)(~10.20Mb),dup(6)(q25.3q27)(~11.52Mb),dup(7)(q11.23q21.11)(~4.80Mb),dup(8)(p12p11.21)(~7.20Mb),dup(9)(q21.33q22.31)(~5.60Mb),dup(9)(q22.31q31.1)(~10.00Mb),dup(19)(q13.32q13.33)(~4.60Mb),+20(~40%),dup(22)(q11.1q11.21)(~4.40Mb) |
| 191 | PT23_2 | PB1+PB2 | -7,-17,+20 | -7,-17,+20 |
| 192 | PT23_3 | PB1+PB2 | +X(~67%),-3,-7,-9(~69%),-10,-12,-13(~69%),-14(~59%),-15(~67%),+18(~59%),-21(~68%) | -3(~68%),-7,-9(~65%),-10,-12,-13(~66%),-14(~63%),-15(~67%),-21(~68%) |
| 193 | PT23_4 | PB1+PB2 | Euploidy | Euploidy |
| 194 | PT23_5 | PB1+PB2 | +16,-21,+22 | +16,-21,+22 |
| 195 | PT23_6 | PB1+PB2 | -19,+21 | -19,+21 |
| 196 | PT24_2 | PB1+PB2 | Euploidy | Euploidy |
| 197 | PT24_2 | PB1+PB2 | Euploidy | Euploidy |
| 198 | PT25_2 | PB1+PB2 | N/A | +17 (contain 10% of Y-chromosome) |
| 199 | PT25_4 | PB1+PB2 | -5,-13,+19,-22 | -5,-13,+19,-22 |
| 200 | PT26_4 | PB1+PB2 | -4,+15,-22 | -4,+15,-22 |
| 201 | PT27_2 | PB1 | -22 | -22 |
| 202 | PT27_2 | PB2 | -19 | -19 |
| 203 | PT27_4 | PB1 | -19 | -19 |
| 204 | PT27_4 | PB2 | +19 | +19 |
| 205 | PT28_1 | PB1+PB2 | +2 | +2 |
| 206 | PT28_2 | PB1+PB2 | +22 | +22 |
| 207 | PT28_3 | PB1+PB2 | Euploidy | Euploidy |
| 208 | PT29_1 | PB1+PB2 | Euploidy | Euploidy |
| 209 | PT29_2 | PB1+PB2 | N/A | N/A |
| 210 | PT29_3 | PB1+PB2 | Euploidy | Euploidy |
| 211 | PT29_4 | PB1+PB2 | dup(14)(q32.31q32.33)(~4.00Mb),dup(19)(q13.32q13.33)(~4.20Mb),-21 | dup(14)(q32.31q32.33)(~4.00Mb),dup(19)(q13.32q13.33)(~4.20Mb),-21 |
| 212 | PT29_5 | PB1+PB2 | Euploidy | Euploidy |
| 213 | PT29_6 | PB1+PB2 | -2,-16 | -2,-16 |
| 214 | PT30_12 | PB1+PB2 | -18 | -18 |

**Supplementary Table S9. Comparison of copy number variant (CNV) results between polar body (PB) and trophectoderm (TE) samples.** This table includes all oocyte CNV results inferred from PBs, corresponding blastocyst CNV results, and CNV origin.

| **Sample ID** | **Type of the samples** | **CNV results of oocyte** | **TE-based PGT-A** | **consistency** | **CNV origin** |
| --- | --- | --- | --- | --- | --- |
| PT02_1 | PB1 | N/A | -4(~52%) | \ | \ |
| PT02_1 | PB2 |  |  |  |  |
| PT03_4 | PB1 | Euploidy | -X | Inconsistent | -X(P) |
| PT03_4 | PB2 |  |  |  |  |
| PT04_2 | PB2 | +15,-16 | +15,-16 | consistent | +15(M),-16(M) |
| PT04_2 | PB1 |  |  |  |  |
| PT04_3 | PB1 | -X | -X | consistent | -X(M) |
| PT04_3 | PB2 |  |  |  |  |
| PT05_3 | PB2 | del(1)(q21.1q21.2)(~1.2MB) | del(1)(q21.1q21.2)(~1.2MB),-4 | Inconsistent | del(1)(q21.1q21.2)(~1.2MB)(M),-4(M:M) |
| PT05_3 | PB1 |  |  |  |  |
| PT06_2 | PB2 | N/A | -17(~64%),+22(~64%) | \ | \ |
| PT06_2 | PB1 |  |  |  |  |
| PT06_6 | PB1+PB2 | Euploidy | Euploidy | consistent | \ |
| PT06_8 | PB1 | -16,+21,+22 | -16,+21,+22 | consistent | -16(M),+21(M),+22(M) |
| PT06_8 | PB2 |  |  |  |  |
| PT07_1 | PB1 | N/A | Euploidy | \ | \ |
| PT07_1 | PB2 |  |  |  |  |
| PT07_3 | PB1 | N/A Contamination | Euploidy | \ | \ |
| PT07_3 | PB2 (Contamination) |  |  |  |  |
| PT07_5 | PB1 (Contamination) | N/A | Euploidy | \ | \ |
| PT07_5 | PB2 |  |  |  |  |
| PT07_6 | PB1 | -11 | -11 | consistent | -11(M) |
| PT07_6 | PB2 |  |  |  |  |
| PT07_7 | PB1 | +5(~52%) | Euploidy | Inconsistent | \ |
| PT07_7 | PB2 (Contamination) |  |  |  |  |
| PT08_4 | PB1 | Euploidy | Euploidy | consistent | \ |
| PT08_4 | PB2 |  |  |  |  |
| PT08_5 | PB1 | Euploidy | +1(~36%),-10(~66%),-18(~63%) | Inconsistent | +1(~36%)(P:M),-10(~66%)(P),-18(~63%)(M:M) |
| PT08_5 | PB2 |  |  |  |  |
| PT08_6 | PB1 | Euploidy | del(2)(q24.3q37.3)(~76.40Mb), del(14)(q31.2q32.33)(~23.15Mb) | Inconsistent | del(2)(q24.3q37.3)(~76.40Mb)(P), del(14)(q31.2q32.33)(~23.15Mb)(P) |
| PT08_6 | PB2 |  |  |  |  |
| PT08_8 | PB2 | Euploidy | Euploidy | consistent | \ |
| PT08_8 | PB1 |  |  |  |  |
| PT09_1 | PB1+PB2 | Euploidy | del(4)(p15.2p15.1)(-6.40Mb) | Inconsistent | del(4)(p15.2p15.1)(-6.40Mb)(P) |
| PT09_4 | PB1+PB2 | Euploidy | -9 | Inconsistent | -9(P) |
| PT09_6 | PB1+PB2 | Euploidy | Euploidy | consistent | \ |
| PT09_7 | PB1+PB2 | Euploidy | Euploidy | consistent | \ |
| PT12_2 | PB1 | -13 | -13 | consistent | -13(M) |
| PT12_2 | PB2 |  |  |  |  |
| PT12_3 | PB1 | -21(10% Y contamination) | -21 | consistent | -21(M) |
| PT12_3 | PB2 (Contamination) |  |  |  |  |
| PT12_5 | PB1 | +21 | +21 | consistent | +21(M) |
| PT12_5 | PB2 |  |  |  |  |
| PT12_6 | PB1 | Euploidy | del(8)(q11.23q24.3)(~92.36Mb,~53%) | Inconsistent | del(8)(q11.23q24.3)(~92.36Mb,~53%)(M:M) |
| PT12_6 | PB2 |  |  |  |  |
| PT13_1 | PB1 | +15 | Euploidy | Inconsistent | \ |
| PT13_1 | PB2 |  |  |  |  |
| PT13_2 | PB1 | Euploidy | Euploidy | consistent | \ |
| PT13_2 | PB2 |  |  |  |  |
| PT13_4 | PB1 | Euploidy | Euploidy | consistent | \ |
| PT13_4 | PB2 |  |  |  |  |
| PT13_5 | PB1 | +13(~67%),+14(~50%),+16 | Euploidy | Inconsistent | \ |
| PT13_5 | PB2 |  |  |  |  |
| PT13_6 | PB1 | Euploidy | Euploidy | consistent | \ |
| PT13_6 | PB2 |  |  |  |  |
| PT14_1 | PB1 | +17 | +17 | consistent | +17(M) |
| PT14_1 | PB2 |  |  |  |  |
| PT14_2 | PB1 | Euploidy | +20（~46%） | Inconsistent | \ |
| PT14_2 | PB2 |  |  |  |  |
| PT14_3 | PB1 | Euploidy | +X(~31%),-Y(~46%),+1(~57%), -2,+5(~56%),+8(~54%), -9,+10(~64%),+11(~56%),+12(~62%) +14,+17(~63%),-19,-20,+22(~60%) | Inconsistent | \ |
| PT14_3 | PB2 |  |  |  |  |
| PT14_4 | PB1 | +16 | -3(~35%).+16(~44%) | Inconsistent | \ |
| PT14_4 | PB2 |  |  |  |  |
| PT14_5 | PB1 | Euploidy | Euploidy | consistent | \ |
| PT14_5 | PB2 |  |  |  |  |
| PT14_6 | PB1 | N/A Contamination | Euploidy | \ | \ |
| PT14_6 | PB2  (Contamination) |  |  |  |  |
| PT15_1 | PB1 | Euploidy | Euploidy | consistent | \ |
| PT15_1 | PB2 |  |  |  |  |
| PT15_2 | PB1 | Euploidy | Euploidy | consistent | \ |
| PT15_2 | PB2 |  |  |  |  |
| PT15_4 | PB1 | -17 | -17 | consistent | -17(M) |
| PT15_4 | PB2 |  |  |  |  |
| PT15_5 | PB1 | Euploidy | Euploidy | consistent | \ |
| PT15_5 | PB2 |  |  |  |  |
| PT15_6 | PB1 | +17, +22(59%) | Euploidy | Inconsistent | \ |
| PT15_6 | PB2 |  |  |  |  |
| PT16_1 | PB1 | N/A | Euploidy | \ | \ |
| PT16_1 | PB2 |  |  |  |  |
| PT16_2 | PB1 | Euploidy | Euploidy | consistent | \ |
| PT16_2 | PB2 |  |  |  |  |
| PT16_3 | PB1 | del(5)(q13.2q35.3)(~109.52Mb), dup(18)(q21.33q23)(~17.08Mb) | dup(2)(p25.3p15)(~63.20Mb,~51%), del(5)(q13.2q35.3)(~109.52Mb), del(17)(p13.3p11.2)(~19.20Mb,~48%), dup(18)(q21.33q23)(~17.08Mb) | Inconsistent | dup(2)(p25.3p15)(~63.20Mb,~51%)（M:SPH), del(5)(q13.2q35.3)(~109.52Mb)(M), del(17)(p13.3p11.2)(~19.20Mb,~48%)(M:M), dup(18)(q21.33q23)(~17.08Mb)(M) |
| PT16_3 | PB2 |  |  |  |  |
| PT16_4 | PB1 | del(5)(q13.2q35.3)(~109.52Mb), dup(18)(q21.33q23)(~17.08Mb), -19 | del(5)(q13.2q35.3)(~109.52Mb), dup(18)(q21.33q23)(~17.28Mb), -19 | consistent | del(5)(q13.2q35.3)(~109.52Mb)(M), dup(18)(q21.33q23)(~17.28Mb)(M), -19(M) |
| PT16_4 | PB2 |  |  |  |  |
| PT16_5 | PB1 | dup(5)(q13.2q35.3)(~109.52Mb), del(18)(q21.33q23)(~17.08Mb) | dup(5)(q13.2q35.3)(~109.52Mb), -8(~48%), del(18)(q21.33q23)(~17.28Mb) | Inconsistent | dup(5)(q13.2q35.3)(~109.52Mb)(M), -8(~48%)(P), del(18)(q21.33q23)(~17.28Mb)(M) |
| PT16_5 | PB2 |  |  |  |  |
| PT16_6 | PB1 | Euploidy | Euploidy | consistent | \ |
| PT16_6 | PB2 |  |  |  |  |
| PT16_7 | PB1 | del(5)(q13.2q35.3)(~109.52Mb),-16, dup(18)(q21.33q23)(~17.08Mb)((10% Y contamination) | del(5)(q13.2q35.3)(~109.52Mb),-16, dup(18)(q21.33q23)(~17.08Mb) | consistent | del(5)(q13.2q35.3)(~109.52Mb)(M), -16(M), dup(18)(q21.33q23)(~17.08Mb)(M) |
| PT16_7 | PB2  (Contamination) |  |  |  |  |
| PT16_8 | PB1 | dup(5)(p15.33q13.2)(~71.80Mb), del(5)(q13.2q35.3)(~109.52Mb), del(18)(p11.32q21.33)(~61.20Mb), dup(18)(q21.33q23)(~17.28Mb) | dup(5)(p15.33q13.2)(~71.60Mb), del(5)(q13.2q35.3)(~109.52Mb), del(18)(p11.32q21.33)(~61.20Mb), dup(18)(q21.33q23)(~17.08Mb) | consistent | dup(5)(p15.33q13.2)(~71.60Mb)(M), del(5)(q13.2q35.3)(~109.52Mb)(M), del(18)(p11.32q21.33)(~61.20Mb)(M), dup(18)(q21.33q23)(~17.08Mb)(M) |
| PT16_8 | PB2 |  |  |  |  |
| PT16_10 | PB1 | del(5)(q13.2q35.3)(~109.52Mb), dup(18)(q21.33q23)(~17.08Mb) | del(5)(p15.33q13.2)(~71.60Mb), dup(18)(p11.32q21.33)(~61.20Mb) | consistent | del(5)(p15.33q13.2)(~71.60Mb)(M), dup(18)(p11.32q21.33)(~61.20Mb)(M) |
| PT16_10 | PB2 |  |  |  |  |
| PT16_11 | PB1 | dup(5)(q13.2q35.3)(~109.52Mb), del(18)(q21.33q23)(~17.08Mb) | X(~41%), dup(5)(p15.33q13.2)(~71.60Mb,~33%), dup(5)(q13.2q35.3)(~109.52Mb), del(18)(q21.33q23)(~17.08Mb) | Inconsistent | -X(~41%)(P), dup(5)(p15.33q13.2)(~71.60Mb,~33%)(P) dup(5)(q13.2q35.3)(~109.52Mb)(M), del(18)(q21.33q23)(~17.08Mb)(M) |
| PT16_11 | PB2 |  |  |  |  |
| PT16_13 | PB1 | -5,+18 | -5,+18 | consistent | -5(M),+18(M) |
| PT16_13 | PB2 |  |  |  |  |
| PT16_14 | PB1 | -18 | -18 | consistent | -18(M) |
| PT16_14 | PB2 |  |  |  |  |
| PT16_15 | PB1 | Euploidy | Euploidy | consistent | \ |
| PT16_15 | PB2 |  |  |  |  |
| PT16_16 | PB1  (Contamination) | N/A Contamination | dup(5)(p15.33q13.2)(~71.60Mb), del(18)(p11.32q21.33)(~61.20Mb) | \ | \ |
| PT16_16 | PB2 |  |  |  |  |
| PT17_1 | PB1+PB2 | Euploidy | Euploidy | consistent | \ |
| PT17_3 | PB1+PB2 | Euploidy | Euploidy | consistent | \ |
| PT17_5 | PB1+PB2 | Euploidy | Euploidy | consistent |  |
| PT17_8 | PB1+PB2 | Euploidy | Euploidy | consistent | \ |
| PT17_11 | PB1+PB2 | Euploidy | del(5)(p15.33q14.1)(~81.40Mb,~31%), dup(9)(q21.11q34.3)(~70.40Mb), del(18)(p11.32q12.3)(-40.40Mb,~36%), -21(~31%) | Inconsistent | del(5)(p15.33q14.1)(~81.40Mb,~31%)(P), dup(9)(q21.11q34.3)(~70.40Mb)(P), del(18)(p11.32q12.3)(-40.40Mb,~36%)(P), -21(~31%)(P) |
| PT17_12 | PB1+PB2 | Euploidy | -8(~34%), dup(18)(q11.1q23)(~59.68Mb,~43%) | Inconsistent | Balanced |
| PT17_13 | PB1+PB2 | Euploidy | del(6)(p25.3p21.1)(-46.00Mb,~41%) | Inconsistent | del(6)(p25.3p21.1)(-46.00Mb,~41%)(M:M) |
| PT17_14 | PB1+PB2 | Euploidy | del(17)(p13.3p11.2)(~21.60Mb,~35%) | Inconsistent | Balanced |
| PT18_2 | PB1+PB2 | Euploidy | Euploidy | consistent | \ |
| PT19_1 | PB1+PB2 | +6(~45%),+10(~45%) | Euploidy | Inconsistent | \ |
| PT23_2 | PB1+PB2 | +7,+17,-20 | dup(7)(p22 3q31.32)(~123.40Mb), dup(7)(q31.32q35)(-20.80Mb), +17.-20 | consistent | +7(M),+17(M),-20(M) |
| PT23_3 | PB1+PB2 | -X(~67%),+3,+7,+9(~69%),+10, +12,+13(~69%),+14(~59%), +15(~67%),-18(~59%),+21(~68%) | +3(-63%)+7+9.+10(-68%),+12(-56%), +13(~61%),+14(63%),+15(-63%), +21(-66%) | Inconsistent | \ |
| PT23_4 | PB1+PB2 | Euploidy | -6(-37%),-7(-47%),+8,+12(-45%), +13(-45%),+14(-42%),+15, +16(~43%),-18(-67%)+22(-60%) | Inconsistent | -6(-37%)(Balanced),-7(-47%)(M:M), +8(Balanced),+12(-45%),(Balanced), +13(-45%)(Balanced),+14(-42%),(Balanced), +15(M:M),+16(~43%)(Balanced), -18(-67%)(Balanced),+22(-60%)(M:M) |
| PT23_5 | PB1+PB2 | -16,+21,-22 | -2,-16, dup(19)(p13.3p12)(-21.40Mb~31%) +21,-22 | Inconsistent | -2(P:M),-16(M), dup(19)(p13.3p12)(-21.40Mb~31%)(Balanced) +21(M),-22(M) |
| PT23_6 | PB1+PB2 | +19,-21 | +19,-21 | consistent | +19(M),-21(M) |
| PT24_1 | PB1+PB2 | Euploidy | Euploidy | consistent | \ |
| PT24_2 | PB1+PB2 | Euploidy | dup(18)(q22.1q23)(~13.60Mb.~61%) | Inconsistent | \ |
| PT27_2 | PB1 | +19,+22 | +19(~59%),+22 | consistent | +19(M),+22(M) |
| PT27_2 | PB2 |  |  |  |  |
| PT27_4 | PB1 | Euploidy | Euploidy | consistent | \ |
| PT27_4 | PB2 |  |  |  |  |
